# Hypervariable antigen genes in malaria have ancient roots

**DOI:** 10.1186/1471-2148-13-110

**Published:** 2013-05-31

**Authors:** Martine M Zilversmit, Ella K Chase, Donald S Chen, Philip Awadalla, Karen P Day, Gil McVean

**Affiliations:** 1 National Institute of Allergy of Infectious Disease, National Institutes of Health, 12735 Twinbrook Parkway, Rockville, MD 20852, USA; 2 Department of Microbiology, NYU Langone Medical Center, 341 East 25th Street, New York, NY 10010, USA; 3 Department of Statistics, University of Oxford, 1 South Parks Road, Oxford OX1 3TG, UK; 4 CHU Sainte-Justine Centre de Recherche, Universit de Montral, 3175 Cote-Ste-Catherine, Montreal, QC, H3T 1C5, Canada; 5 Wellcome Trust Centre for Human Genetics, Roosevelt Drive, Oxford OX3 7BN, UK

**Keywords:** Non-allelic homologous recombination, Hidden Markov-model, *var* genes, Malaria, PfEMP1, Gene family evolution, Balancing selection

## Abstract

**Background:**

The *var* genes of the human malaria parasite *Plasmodium falciparum* are highly polymorphic loci coding for the erythrocyte membrane proteins 1 (PfEMP1), which are responsible for the cytoaherence of *P. falciparum* infected red blood cells to the human vasculature. Cytoadhesion, coupled with differential expression of *var* genes, contributes to virulence and allows the parasite to establish chronic infections by evading detection from the host’s immune system. Although studying genetic diversity is a major focus of recent work on the *var* genes, little is known about the gene family's origin and evolutionary history.

**Results:**

Using a novel hidden Markov model-based approach and *var* sequences assembled from additional isolates and species, we are able to reveal elements of both the early evolution of the *var* genes as well as recent diversifying events. We compare sequences of the *var* gene DBLα domains from divergent isolates of *P. falciparum* (3D7 and HB3), and a closely-related species, *Plasmodium reichenowi*. We find that the gene family is equally large in *P. reichenowi* and *P. falciparum* -- with a minimum of 51 *var* genes in the *P. reichenowi* genome (compared to 61 in 3D7 and a minimum of 48 in HB3). In addition, we are able to define large, continuous blocks of homologous sequence among *P. falciparum* and *P. reichenowi var* gene DBLα domains. These results reveal that the contemporary structure of the *var* gene family was present before the divergence of *P. falciparum* and *P. reichenowi*, estimated to be between 2.5 to 6 million years ago. We also reveal that recombination has played an important and traceable role in both the establishment, and the maintenance, of diversity in the sequences.

**Conclusions:**

Despite the remarkable diversity and rapid evolution found in these loci within and among *P. falciparum* populations, the basic structure of these domains and the gene family is surprisingly old and stable. Revealing a common structure as well as conserved sequence among two species also has implications for developing new primate-parasite models for studying the pathology and immunology of falciparum malaria, and for studying the population genetics of *var* genes and associated virulence phenotypes.

## Background

Despite forty years of research, there is no effective vaccine against malaria, which caused an estimated 655,000 deaths in 2011, almost all of which are due to falciparum malaria [1]. Notably, however, people from endemic areas are able to develop natural adaptive (but not purifying) immunity by adulthood after repeated infections [2]. One reason why the etiological agent of this form of malaria, the parasite *Plasmodium falciparum*, can evade the human immune system is the very high level of sequence diversity in the *Plasmodium falciparum* Erythrocyte Membrane Proteins 1 (PfEMP1s), expressed on the surface of infected red cells. These proteins can provoke an immune response and are known virulence factors, contributing to the adherence of infected erythrocytes to the vascular endothelium, clogging capillary vasculature in the brain and body [3]. PfEMP1s are encoded by a rapidly evolving and large multigene family (ranging from at least 40 to over 60 genes per genome), the *var* genes. These genes evolve extremely rapidly, to the point where they do not have stable locations in the genome [4]. Because of this mobility, combined with a very high level of sequence diversity [5], these genes cannot be described as having a conventional structure of shared alleles among populations. Recombination, combined with single-base mutation, is the mechanism of rapid evolution that generates the diversity at these loci [4,6,7].

Unlike the other well-studied antigens in *P. falciparum* (e.g. circumsporozoite protein, the Merozoite Surface Proteins-1, -2, and -3, and Apical Membrane Antigen-1), *var* genes are not shared among all human malaria parasites. Only a few *var* genes have ever been found in another species, the closely related chimpanzee parasite *P. reichenowi*[7], suggesting that the large and complex *var* gene family is unique to *P. falciparum*.

A recent origin for these genes would be highly unusual given what is known about the evolution of the other important *Plasmodium* antigen-coding genes. Alleles at these loci in *P. falciparum* populations are frequently very old, predating the divisions of the populations and tend to be older than other loci and alleles in the genome [8,9]. Balancing selection acts on loci for which diversity is advantageous because of frequency dependent selection. The result of this type of selective force is that old alleles, which would typically be lost in a species or population over time, are maintained because they become more beneficial to the organism as they become rarer, hence selection drives them up in frequency again. This is the case with antigens in pathogens of vertebrates like malaria parasites, because the host’s immune system is more effective against proteins it is exposed to more frequently. As a result, parasites expressing less-common proteins avoid detection more effectively.

In spite of the importance of the *var* genes and extensive studies of their diversity [5,10], the early evolution of the *var* genes has been difficult to examine because frequent recombination among paralogs renders traditional methods for examining evolution, such as phylogenetic inference, ineffective. Earlier studies have shown that mosaic recombination plays an important role in the evolution of the *var2csa* gene [11,12]. This gene is unique in the genome, distinct from the paralogs that code for PfEMP1s [13], and only recombines homologously. However, it is structurally related to the larger *var* gene family examined here. Because the *var* genes do not have a stable genome location or recombine in a straightforward homologous nature like the *var2csa* genes or other antigens in *P. falciparum*[8], specialized methods are required to examine their evolutionary history. Here we have developed a statistical method using a hidden Markov model to study the evolutionary relationships of the *var* genes and the early evolution of this complex gene family. Although these genes are known for extreme diversity and rapid recent evolution, we demonstrate an older history for this gene family than was previously shown, whereby recurrent shuffling of ancient semi-conserved sequence blocks underlies sequence diversity.

## Results

### *var* genes in Laverania: the structure of the gene family

*Plasmodium falciparum* and *P. reichenowi* are the only two species of the recently resurrected subgenus *Laverania* with available genome sequence data. Although our knowledge of the evolution and structure of the *P. falciparum* genome remains incomplete, even less is known about the genetics and genomics of *P. reichenowi.* Reconstructing the *var* genes in *P. reichenowi* allows for a comparative analysis and improved evolutionary understanding of these genes, which function both as virulence factors and in transmission [14].

We have annotated the available capillary sequence data of the *P. reichenowi* genome for *var* genes, using a prototypical sequence for the DBLα binding domain [15] as the model. The DBLα binding domain, averaging 1.8 kb in length, is used as the homologous region for comparison to define *var* genes in this study because it is the only functional domain common to all *P. falciparum var* gene paralogs.

Using the tBLASTx algorithm and the prototype DBLα domain, we were able to extract reads (typical length 900 bp) from the Sanger database [http://www.sanger.ac.uk/resources/downloads/protozoa/plasmodium-reichenowi.html]. Using the Clean Data assembly algorithm in Sequencher (GeneCodes), 51 unique DBLα regions in *P. reichenowi* were recovered (including those for the comparatively conserved *var1csa* and *var2csa* genes). This minimum number of *var* genes in *P. reichenowi* is within range of that for a *P. falciparum var* gene family*,* thus demonstrating that the family is equally large in both species. These assembled loci include some that appear to be pseudogenized by frameshifts, although this may represent sequencing errors.

**Figure 1:**
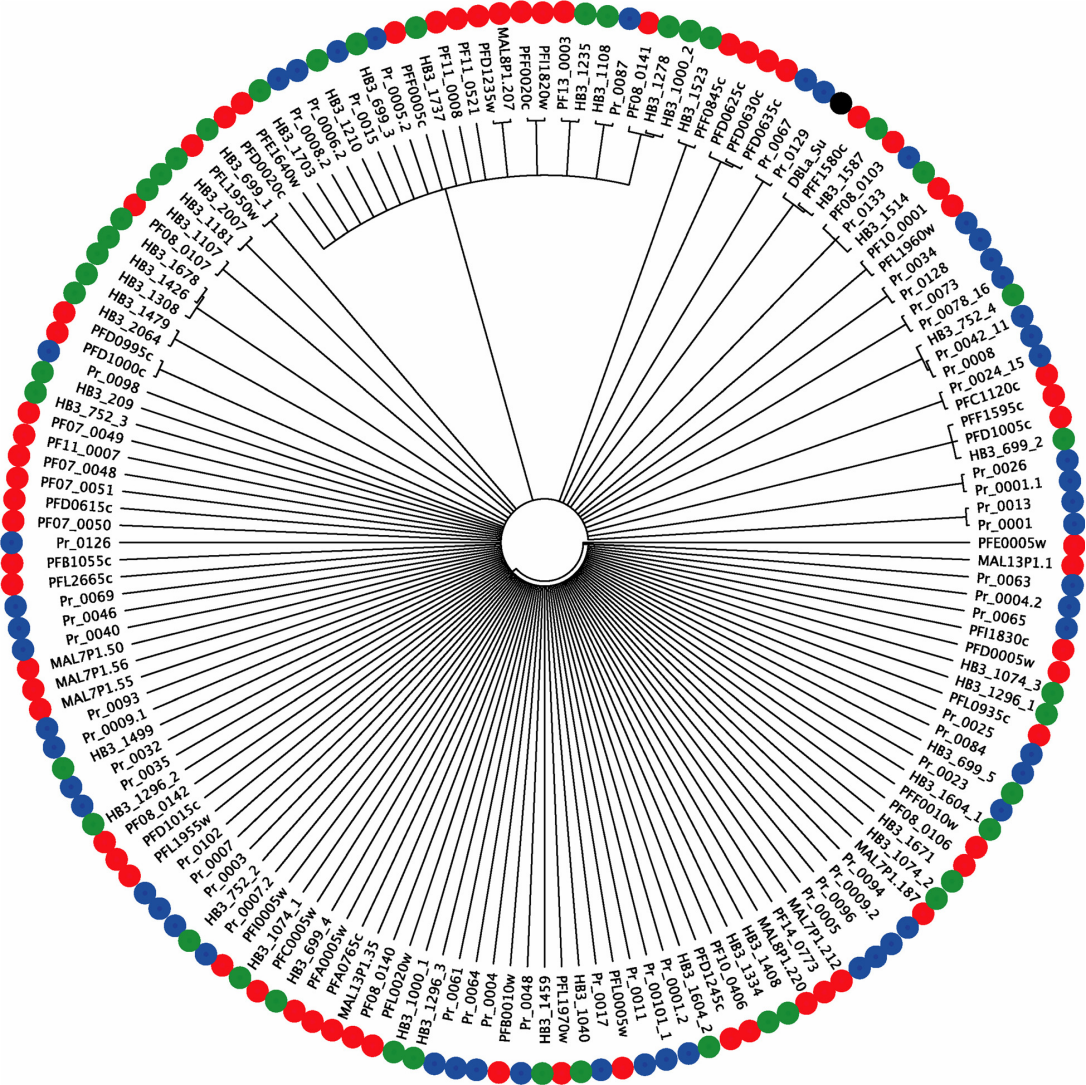
**A proportional (branch-lengths are not informative) maximum-likelihood phylogeny of amino acid sequences of all DBLα sequences for *var *genes in *P. falciparum *isolates 3D7 (PF and MAL, red circles) and HB3 (green circles), and *P. reichenowi *(Pr, blue circles), including the DBLα region used for reference in this study (DBLa_Su black circles).** The tree is a consensus of 1000 bootstrapped replicates, showing only nodes represented in ≥ 70% of the replicate trees.

Phylogenetic analysis of the *var* gene DBLα domain reveals that the *P. reichenowi var* genes do not cluster together (Figure 1). Rather, the *var* genes in the analysis, from *P. reichenowi* and two divergent *P. falciparum* isolates (representing the Old World (3D7), and the New World (HB3)), form a diffuse set of small groupings with very little support in the basal branches of the tree. This result indicates that the *var* genes likely arose as an entire family before the *P. falciparum-P. reichenowi* speciation event 2.5-6 million years ago [16]. Such an unresolved tree typically indicates rapid evolution by point mutation (saturating the informative sites), reticulate evolution by recombination such that a bifurcating tree diagram cannot represent the evolution, or a combination of these two mechanisms.

### New methods for exploring the evolution of rapidly evolving gene families

**Figure 2:**
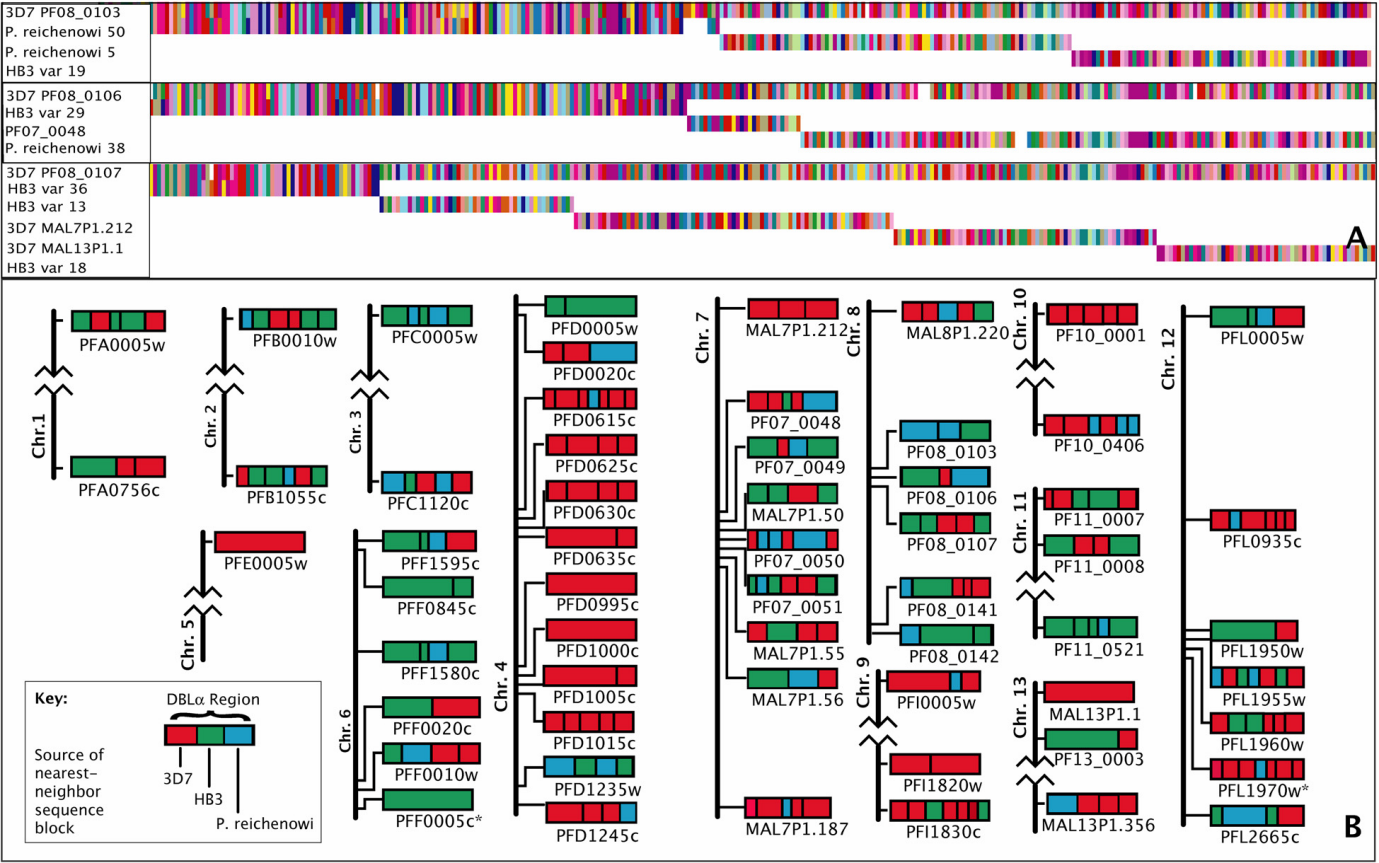
**An example of the output and results of the analytical method.** (**A**) Amino acid alignments are shown for three exemplary target sequences used in the analysis (the first sequence in each alignment is the target) used in the analysis. Each destination sequence is shown for the fragment (for recombinant sequences) or whole sequences (for non-recombinant results, in these examples) that showed the homology match. Each color represents a different amino acid. (**B**) Mosaic ancestry of the 3D7 DBLα domains. Schematic representations of domains from the 3D7 genome showing the source of the nearest-neighbor of each homology block: 3D7 (red, within genome match, recent homology), HB3 (green, within species match, older homology) and *P. reichenowi* (blue, across species match, ancient homology). Homology regions are separated by black vertical lines, and adjacent blocks of the same color represent blocks originating from different genes of the same genome. Genes are placed in genomic context showing relative locations to each other and to the telomeres of each chromosome (chromosomes drawn roughly to scale). Possible pseudogenes are marked with *. Only robust, high-scoring alignments are shown. Alignments for 3D7 PF08_0140 and 3D7 PFL0020w are not shown due to poor scores.

The poor performance of phylogenetic and other methods in reconstructing the history of the *var* genes points to the unusual evolution of these genes. Thus, new methods are necessary to identify relationships between sequences. To this end, we have developed a means to examine the homologous relationships among orthologous and paralogous sequences allowing for recombination*.* This statistical method, *Tesserae,* uses a hidden Markov model to find homology, a product of approximate conditionals (PAC) likelihood [17] to estimate the recombination parameter, a modified global alignment algorithm (Needleman-Wunsch) to detect mosaic recombination [18], and the BLOSUM62 matrix is used for amino acid transitions. (Figure 2A shows an example of the output of the program, see Additional file 1 for details of the method and the model). This model-based approach aims to reconstruct each sequence in a data set as an imperfect mosaic of one or more donor sequences, allowing for substitutions, insertions, deletions, and recombination-induced mosaicism. The parameters for insertions, deletions and mutations are estimated by expectation-maximization (Baum-Welch algorithm) with no recombination (a conservative approach for detecting the effects of recombination). Subsequently, a composite-likelihood surface for the recombination parameter is calculated and, for the maximum-composite-likelihood parameter estimates, the maximum likelihood path through the HMM is calculated for each sequence (see Additional file 1). The absolute value of the likelihood, normalized for sequence length, is used as the metric by which alignment robustness is measured. The cutoff was established by training the system on known recombination breakpoints and regions known to be without recombination, discussed below. This last step is only necessary for gene families, such as the *var* genes, whose extremely rapid evolution makes identifying orthologous loci among individuals difficult.

The algorithm was tested using both simulated and empircal data. For simulated data we constructed amino acid sequences of 150 residues in length, in groups (gene families) of 60. Ten “gene families” were generated for each of eight sets of parameters (described in detail in the Additional file 1), with a different level of recombination and gene conversion, or insertion/deletion events, in their history. In the gene families with indels, the algorithm accurately revealed this in the estimation of an appropriately large gap extension parameter in these data, and not in data sets simulated without a history of indels. Gene families with the highest recombination rates revealed some spurious gap extension, but far less than in actual indel data (Additional file 1: Figure S2). The algorithm estimates a very low level of recombination for datasets with no recombination or indels (some bias is expected since the recombination rate must be positive), and increasingly high recombination rates for gene families simulated with comparably higher rates (Additional file 1: Figure S3). Precise calculations of the recombination rates were possible due to the controlled creation of these artificial sequences using the coalescent. Comparisons of the exact values to those calculated by the algorithm showed a high level of accuracy in the estimated recombination rates (see Additional file 1 for a detailed description of the simulation protocols and results). In addition, the difference in likelihood between models with and without recombination for each sequence was also assessed, with the statistical significance of the improvement of recombination models over those without as P < 1 × 10^-32^.

The algorithm was also tested against empirical recombining and non-recombining data, both for reciprocal exchange (in the B and C group *var* genes [19]) and gene conversion (the Acyl CoA-Synthetase gene family [20] and the *var* genes (PFD0625c/PDF0635c and PFD0630c/PFD0635c [21])). To test for false positives, we used a non-recombining data set, 96 complete mitochondrial genomes from *P. falciparum*[22]. The program was able to accurately recover all known recombination events, while finding no recombination history in the mitochondrial data, as expected.

Using *Tesserae* with sequence data from across individuals (or isolates in the case of pathogens) and species, it is possible to infer the relative rates of different evolutionary processes, because the source of the donor sequences reveals the relative age of the recombination events or the homologous sequence. Donors from the same genome indicate recent events, those from a divergent individual of the same species reveal events occurring since speciation, and between-species matches represent ancient homology and old recombination events.

With *Tesserae,* we find high-scoring alignments for all but a small minority of loci, revealing aspects of the evolutionary history of each *var* locus used in the analysis. Results for the DBLα domains in 3D7 are shown (Figure 2B). These results reveal extensive mosaic recombination at the majority of loci.

Although some within-genome, and thus recent, gene duplicates are recovered using this method (e.g. PFD0995c and PFD1000c on chromosome 4, Figure 2B), older mosaicism is widespread, with 31 of the 58 loci showing sequence that is conserved between species, in 39 sequence blocks. Both *P. falciparum* clones show 13.3% of the sequence blocks preferentially matched outside of the species, with *P. reichenowi* (Table 1). In addition, roughly 30% of the sequence of each isolate’s DBLα sequence blocks shows preferential matching within the genome itself (self-matching) (Table 1).

**Table 1:** Percent recombination blocks shared among genomes

	***P. reichenowi***	***3D7***	***HB3***
***P. reichenowi***	52.2	26.7	21.1
***3D7***	13.3	37.6	49.0
***HB3***	13.3	60.8	25.9

To examine whether the cross-species matches represented older recombination and conserved sequence, or an introgression through recent recombination, we ran the analysis with three additional sets of *P falciparum* DBLα domains from three Old World isolates, that have diverged from 3D7 after speciation (Dd2 (Lao/Indochina), IGH (India), and “PfClin” (Ghana) [23]. If the *P. reichenowi* sequence that matched 3D7 represented a recent introgression and subsequent recombination, then these homology regions would remain the best match, even with more recently diverged sequence present (the matching between species would not be reduced in frequency). However, if the conserved sequence blocks represent recombination occurring before speciation, the recombination blocks in the DBLα domains of 3D7 would preferentially match with the other Old World isolates, which had separated more recently and therefore would have a higher degree of similarity. Running the analysis with the additional sequence options for matching, 11 loci in 3D7 have best matches to *P. reichenowi* sequences in 15 blocks (rather than 31 loci in 39 blocks, without these alternate sequences present), with 6.5% of the sequence blocks matching outside the species now (5% for HB3), reduced from 13.3%.

In further examination of the *P. reichenowi* and *P. falciparum* DBLα domain homology regions, we found multiple regions of particularly high homology, classified here in two groups: core motifs and conserved peptides. The core motifs are defined as regions of 100% sequence identity that are found in at least one *P. reichenowi* gene matching two or more from *P. falciparum* and correspond to one of the five known regions of high homology that characterize the DBL domains (termed “homology blocks” by Rask *et al*. [23]). These regions are numbered HB1-5 and are between 18 and 28 residues in length [23]. We recovered 18 sequences corresponding to the conserved motifs, between 10 and 29 residues long, in 36 different *P. reichenowi var* genes and shared with 85 of those from 3D7 and HB3 (details of all loci with each identical motif are available as Additional files 2 and 3). The HB2 motifs are the most frequently represented in this group, and found in approximately half of all loci. The HB3 motifs are found in two different forms in about a third of the DBLα domains, and HB5 are in about one fifth. The HB4 motif had only one identical form match two *P. falciparum* domains (one each in 3D7 and HB3), and the HB1 motifs were not present in these results.

In addition to these core motifs, we also found 53 regions that we describe as conserved peptides, determined by pairwise matches of high identity/similarity between *P. reichenowi* and *P. falciparum* DBLα domains. These peptides range between 24 and 140 residues long, and between 70% and 100% identity (identical amino acids) and 80% to 100% similarity (amino acids with the same or similar functional characteristics). The conserved regions are drawn from 48 non-redundant matches, with the five additional peptides each drawn from a smaller portion of a conserved peptide, with even higher identity. While many *P. reichenowi* loci revealed none of this highly conserved sequence, some loci were represented in multiple matches, the most frequent being Preich_004 with 6 non-redundant regions plus one additional sub-region of high *P. reichenowi*/*P. falciparum* homology. (The full sequences of all the *P. reichenowi* core motifs and conserved peptides, plus the alignments and matching loci from *P. falciparum* are included in Additional files 2, 3, and 4).

**Figure 3:**
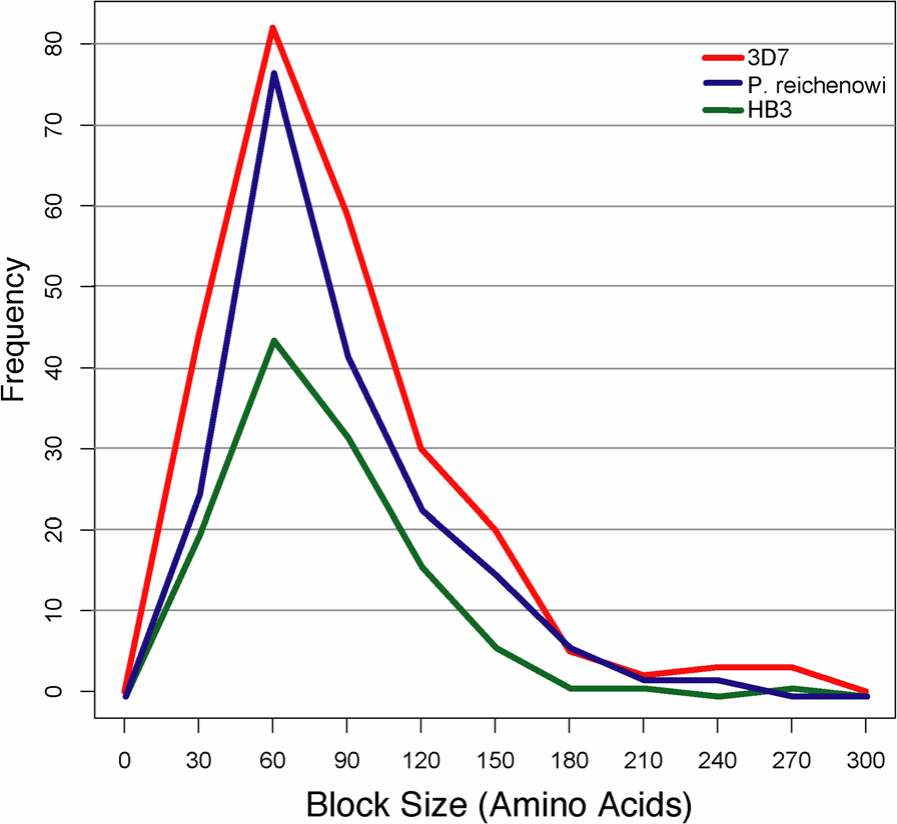
**Distribution of recombination-block sizes.** Both species and all three genomes explored in this study have the same range and mean (11) for distances between recombination breakpoints, which define recombination blocks.

**Figure 4:**
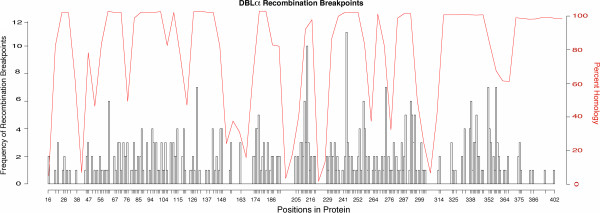
**Our analysis shows that recombination is uniform throughout the DBLα domains and does not show a hot- or coldspot structure.** This lack of structure is revealed in this histogram showing recombination breakpoint frequency at each amino acid position mapped onto a multiple sequence alignment (white bars). This is overlaid with levels of homology (percent of gaps at each position) at each site (red line). Levels of homology are highly inconsistent over the length of DBLα domains and regions that are highly homologous and align well have no gaps, whereas regions with low homology require many gaps. This figure shows that, correcting for regions where there are large gaps (low homology), there are no large increases or decreases in frequency of recombination breaks typical of hot- or coldspot structure. The multiple sequence alignment is of all DBLα domains from all samples used in this study.

Our results reveal that recombination of homology regions is shaping the *var* gene repertoires in the same way within and among species. This recombination, however, does not reveal a particular hot- or coldspot structure along the DBLα domains. Table 2 and Figure 3 show that the average distance between breakpoints, and the variance of these distances, is the same in all samples studied. The high variance in block size also reveals the lack of hot- or coldspots of recombination in the DBLα regions. The homogeneity of recombination rate is also evident in a mapping of the positions and frequency of recombination breakpoints along the protein sequence in an alignment, showing recombination breaks at almost every residue (Figure 4). Due to insertions and deletions within the DBLα domains, mapping on an alignment is necessary to ensure homologous positions are used. These insertions and deletions are in low-homology portions of the domain and form large, gapped regions in the alignment between areas of high homology. Correcting for these gapped regions in the alignment shows that the frequency of recombination largely reflects only the number of homologous sequences (or gaps) at that position (Figure 4).

**Table 2:** Size and number of recombination blocks in each sample

**Homology regions**	**Block length**	**No. of blocks**
***P. reichenowi***		
Mean	76.23	4.00
SD	55.95	1.40
**HB3**		
Mean	77.75	3.98
SD	64.79	1.89
**3D7**		
Mean	76.77	4.05
SD	61.04	1.77

## Discussion and conclusions

The similarities in size, structural organization, and sequence diversity demonstrated here for the *var* genes between *P. falciparum* and *P. reichenowi* establish a new understanding of the evolutionary history of this gene family. The work presented here reveals not only the existence of homologous sequence between *P. falciparum* and closely-related species, but also a notable amount of contiguous sequence that is shared (including exact matches for the core motifs that define the DBLα domains), and preservation of the size and structure of the gene family itself. We also elucidate the unusual manner by which these genes evolve, combining recombination with point mutation and birth-death evolution, to create new loci out of older sequence blocks. These findings have implications for understanding the evolution of infectious disease including host-parasite co-evolution, and for expanding new great-ape model systems to study malaria pathology.

### Methods for studying hypervariable paralogs

Previous studies have shown that there is some *var* gene sequence shared among *P. falciparum* and *P. reichenowi*[7,23], and that recombination is likely to contribute to diversifying the genes in *P. falciparum*[6,23]. Here we show the structure of this recombination, and large blocks of sequence, are shared between *P. falciparum* and *P. reichenowi*. We also reveal that these ancient elements are more numerous and more highly conserved than previously thought. These results suggest that the *var* genes are under similar balancing selection pressures evident in other antigens found among all human *Plasmodium* parasites (e.g. circumsporozoite protein, the Merozoite Surface Proteins-1, -2, and -3, and Apical Membrane Antigen-1) [9]. What is unusual about this gene family is how it is evolving. Although recombination appears to be an important mechanism for creating antigenic diversity at many loci in the *P. falciparum* genome [24] and at the *var* genes specifically [6], the recombination here is distinct in its high frequency, evidenced by the large number of breakpoints in the DBLα domains when compared to other antigenic genes [24], and by the non-allelic exchange among paralogs.

Our method of *var* gene recovery from the *P. reichenowi* genome has notable advantages over alternative approaches. Some *var* genes have likely been missed in this analysis because of the level of genome coverage and any partial genes for which the DBLα has been deleted will not be detected. However, we compensated for the overall low-coverage by ensuring the domains had an average of 6X coverage in our regions of interest. (Some loci remained at partial low-coverage, although they were long enough to show that they were unique; this is indicated in the notes of the sequences listed in the Additional file 5). This techinique has advantages over searching assembled contigs (or even genomes), or using short-read data, even at higher coverage. A starting dataset of raw shotgun reads will be less biased towards recovering loci in regions of lower variation and/or recombination, which is the problem with searching assembled contigs or even the genome sequences of *Plasmodium* parasites available at this time. This problem arises due the difficulty in assemblying fast-evolving regions, such as the subtelomeres of each chromosome. Also, assembly of short-read data produced from next-generations sequencing, as opposed to these long-read capillary data, is problematic in regions where the units of recombination may be equal to or smaller in size than the reads themselves, requiring much higher coverage for confident assembly. Finally, data available from shotgun sequence, such as the reads we used, will reveal a much less biased set of *var* genes than those revealed by targeted PCR [5,7].

### How *var* genes evolve: recombination, balancing selection, and birth/death evolution

The findings presented here build on the existing body of work aimed at understanding the structure and evolution of the *var* genes. These adhesive proteins are expressed on the red-cell surface and allow malaria-infected cells to adhere to the tissue of the vascular endothelium [3], out of the peripheral circulation and consequently out of the reticuloendothelial system. Because the PfEMP1s are displayed on the surface of infected cells where they are detectable by the host, maintaining diversity at these loci allows the parasites to evade detection by the human immune system.

Previous work has shown sequence homology exists between *P. falciparum* and *P. reichenowi var* genes [7] and results of the present study extend these findings by demonstrating the similarity in gene family size, DBLα domain structure, and conserved motifs in the DBLα domains. These shared elements show that few characteristics of these genes are unique to *P. falciparum.* By examining the functional region common to all *P. falciparum var* genes, the DBLα binding domain, we find that there are at least 51 *var* genes in *P. reichenowi*, a similar number to what would be expected from this large gene family in *P. falciparum* (approximately 50–60 per genome)*.* These *P. reichenowi* DBLα domains retain the same structure as those identified in *P. falciparum*, with a series “homology blocks” [23] containing sequence that has remained identical between the species over evolutionary time. These results, coupled with those that show that half of the genes in a single genome reveal an element of transpecies ancestry (preferentially over intraspecific origins), shift our understanding of these genes as being composed of sequence that is solely rapidly evolving. Here we show that the unusual elements of the *var* genes are not the recent origins of the genes themselves or the size of the gene family, but the way they have evolved such that older sequence recombines among paralogs (non-allelic homologous recombination) to encode novel antigenic proteins. If the majority of the sequence in the DBLα domains was very new (fast-evolving), the signal of homology among species would have been lost since the split between the human and chimpanzee parasite lineages 2.5 to 6 million years ago [16]. The maintenance of ancient diversity shared among the gene copies in divergent genomes likely points to the ongoing arms-race between the pathogen and the host’s immune response. These results indicate the existence of a form of balanced polymorphism that is giving these blocks of sequence old roots relative to other loci in the genome. Under standard evolutionary forces, we expect that alleles in a population to trace their origins to the time of speciation, or subsequent events [25]. The preservation of ancient diversity, although rare for standard loci in a genome, has been found to be common for other antigenic proteins in malaria parasites [8,9].

Selective pressures for increasing diversity at antigenic loci can maintain sequence morphs from common ancestral species that would normally be lost after speciation. This phenomenon occurs because, as the sequences decline in frequency over time after speciation, they become more advantageous for the individuals that carry them. This frequency-dependent selection then drives the sequence (normally alleles, but in this case the sequence blocks) back up in frequency. The host’s immune system, the selective pressure in this case, is more sensitive to antigens that are more common and, as a result, parasites expressing rarer versions of a sequence have an improved chance of surviving immune detection by the host. This immune selection makes a rare sequence more beneficial.

Balancing selection of this type also has been shown to occur at immune-associated loci in vertebrates. It is notable that these immune loci are coevolving with antigenic proteins, such as the PfEMP1, in order to detect them for the vertebrate host. Trans-species alleles have been maintained at the MHC loci (critical for mammalian immunity to infections disease) of great apes over evolutionary time by balancing selection, with recombination known to be key in diversification of the human alleles [26]. Human MHC loci, when compared with those of chimpanzees, show the same trans-species mosaicism maintained by recombination that is revealed here among the *var* genes. In the MHC, however, this gene family recombination is happening at a notably lower frequency, with very little non-allelic exchange, and only in the loci associated with the most extreme levels of diversity [unpublished observations, Zilversmit, McVean, and Awadalla]*.* Therefore the maintenance of such old sequence in these two species of *Plasmodium* is likely due to host-parasite coevolution. It is important to distinguish the mode of evolution of the *var* genes, however, because it is not an entire gene, or even an entire domain, that is being maintained. Rather it is a continuous section of sequence located within the DBLα domain that is being maintained, which can be exchanged among paralogs as a unit by recombination.

Although recombination in clearly frequent among the DBLα domains, our analysis reveals no hotspot structure for the type of intradomain non-allelic homologous recombination examined, recombination at homologous loci among different paralogs. However, DBL recombination hotspots in the *var* genes have been found [23]. The structure of *var* genes consist of more than just the DBLα domain, rather they are a series of DBL domains (e.g. DBLβ and DBLγ) interspersed with CIDRs (cysteine-rich inter-domain regions). The DBLα domain is unique in that it is the only domain that all *var* genes paralogs have in common (and in the same position in the gene), however each gene has multiple different DBL domains of the other types. Hotspots do occur when interdomain DBL recombination is considered [23], e.g. between DBLβ and DBLγ, but do not appear to be present in intradomain DBLα recombination. The difference in the structure of recombination within domains as opposed to between them is likely due to homology. The DBLα domains are orthologous (homologous due to common ancestry) in this context and have more sequence similarity throughout than an interdomain comparison where the relationship among the domains is more like that of paralogs. As a result, the paralogs have more unevenly distributed regions of higher similarity among each other and these portions of sequence are more likely to be nucleation points for recombination breaks.

The results presented here show that the *var* genes are evolving through a combination of birth-death evolution, point mutation, and rapid and unstructured mosaic recombination. The point mutation is evident in the regions of high amino acid similarity, because these regions diversify by changing codons and thus the amino acid identity, while likely retaining much if not all of the function of the regions. This may allow for a level of sequence diversity that can evade immune recognition without large changes to the protein structure [27]. Birth-death evolution is a common mechanism of gene family evolution, characterized by the continual duplication and deletion of paralogs through unequal crossing over during recombination, evidenced in the *var* gene family by the variability in the paralog number among isolates, and in the apparent recent duplications revealed in the self-genome matches of some DBLα domains in this analysis (e.g. PFD0995c and PFD1000c on chromosome 4, Figure 2B).

### Future directions: population genetics and new primate models of malaria

To fully explore the impact of this new understanding of the *var* genes, detailed comparative studies are necessary comparing the genomes of other species closely related to *P. falciparum*, as well as examining whether the same evolutionary dynamics are evident at the population-level. Multiple new species of malaria parasites have been recently discovered to be closely related to *P. falciparum,* notably *P. gaboni*[28] and an estimated 10 other new *Plasmodium* species of Great Apes [16,29] grouped into the resurrected subgenus *Laverania*. Our results here reveal that many of these new species are likely to have a full suite of *var* genes, because these parasites are shown to be more closely related to *P. falciparum* than *P. reichenowi*. At what point these genes arose in the *Laverania* lineage is an important evolutionary question that can only be addressed by comparative genomics among these new species.

This study was designed to examine the early evolution of the *var* genes. The same methods can be extended to examine the influence of recombination and the balanced maintenance of ancient diversity in their recent evolution. These studies can be accomplished by looking at microevolutionary patterns using population-level data. Although there is an abundance of data on *var* genes from field isolates [5,10], these data remain largely unexplored because the correct tools to disentangle the complex histories of these genes, such as the *Tesserae* program, did not exist. It is possible that by identifying highly conserved regions in field isolates, scientists may be able to trace the frequency with which *var* genes recombine in nature, examine correlations of conserved markers with virulence phenotypes in the parasites, and explore the migration of new paralogs the way drug resistance alleles have been tracked from their originating populations as they spread throughout the world.

## Methods

### DBLα sequence assembly

#### *P. reichenowi*

DNA sequences for DBLα domains were assembled using approximately 900 reads and contigs from the CDC1 sample of *P. reichenowi* from the Wellcome Trust Sanger Institute [http://www.sanger.ac.uk/resources/downloads/protozoa/plasmodium-reichenowi.html]. These reads were retrieved with the tBLASTx algorithm using the DBLα domain from Su et al. 1995 [15] as a reference. The reads and contigs were then assembled and edited using Sequencher v.4.2.2 software (Gene Codes Corporation, MI), with at least 6X coverage necessary to call a region (although some DBLα regions with partial low coverage were used if the regions proved to be unique). This method retrieved some DBLß domains as well, these were not used in the analysis, but ensured that an exhaustive search for DBLα domains was completed.

The DNA sequences were then converted to protein using MacClade software version 4.06 [30]. Additional alignments were generated using CLUSTALW2 software [31] with a BLOSUM30 matrix, a gap opening penalty of 10.0, and gap extension penalty of 2.0 were used to filter for redundant domains.

#### P. falciparum

DBLα domains were identified using CLUSTALW2 alignments as above from whole *var* gene sequences using the domain from Su et al. 1995 [15] as a reference. These whole gene protein sequences were retrieved from PlasmoDB version 4.0 (for the isolate 3D7) and from sequence referenced in S. M. Kraemer *et al*., 2007 [4], supplied by the authors (for HB3). The whole *var* gene sequences were edited to the DBLα domains using MacClade software version 4.06 [31].

### Phylogenetic analysis

All DBLα amino acid sequences in this study were aligned using MUSCLE [32] in Full mode using 16 iterations. The phylogeny was constructed using PhyML [33] with the LG [34] model, NNI tree topology search, and a BioNJ starting tree. 1000 trees were made with bootstrapped data and a consensus tree of all nodes appearing in 70% or more of these trees was made using Phyutility [35].

### The *Tesserae* program

The *Tesserae* program implementing the HMM is written in C language and is available from the authors on request along with a document on how to install and use the program.

## Competing interests

The authors have no competing interests.

## Authors’ contributions

MMZ, GM, and KPD conceived of the original concept. MMZ and GM conceived of and carried out the experiments and prepared the manuscript. EKC and GM constructed the hidden Markov model. DSC contributed to the analysis, experimental concept, and manuscript preparation. KPD and PA contributed to the experimental concept and manuscript preparation. All authors read and approved the final manuscript.

## Supplementary Material

Additional file 1 **Hidden Markov-Model to Recover Mosaic Recombination.** A PDF file showing the full hidden Markov model, in detail, and added information on tests using simulated data. Click here for file

Additional file 2 **Analysis and Results of Core Motifs and Conserved Peptides.** An MS Word document describing and summarizing the analysis of core motifs and conserved peptides between *P. reichenowi* and *P. falciparum*. Click here for file

Additional file 3 **Core motifs (conserved motif classification) and genes with each one.** Core motifs ordered alphabetically, with the listing of all loci with that motif. Click here for file

Additional file 4 **Alignments of conserved peptides.** An MS Word document showing all alignments between *P. reichenowi* and *P. falciparum* for conserved peptides. Click here for file

Additional file 5 ***Plasmodium reichenowi *DBLα Domains.** A tab delimited file containing curated *Plasmodium reichenowi* DBLα domains used in this research. Click here for file
