# Supplementary material for: Hypervariable antigen genes in malaria have ancient roots
Source: BMC Evol Biol. 2013 May 31;13:110. doi: 10.1186/1471-2148-13-110 (PMC3680017; doi:10.1186/1471-2148-13-110)
Supplement: Additional file 1 — Hidden Markov-Model to Recover Mosaic Recombination. A PDF file showing the full hidden Markov model, in detail, and added information on tests using simulated data. [file 1471-2148-13-110-S1.pdf]

Supplementary methods:
Hypervariable antigen genes in malaria have ancient roots

Martine M Zilversmit (1,2,3), Ella K. Chase (2), Donald S. Chen (1), Philip Awadalla (3), Karen P. Day (1), Gil McVean (2)

1- Department of Medical Parasitology, NYU Langone Medical Center, 341 East 25th Street, New York, NY 10010, USA

2 - Department of Statistics, University of Oxford, 1 South Parks Road, Oxford, OX1 3TG, United Kingdom

3 - CHU Sainte-Justine Centre de Recherche, Universit de Montral, 3175 Cote-Ste-Catherine, Montreal, QC, H3T 1C5, Canada

Hidden Markov-Model to Recover Mosaic Recombination

Model construction

We combine two previous probabilistic models for sequence evolution; the pair-HMM used to model pairwise sequence alignments (described in Durbin et al. 1998) and the model of Li and Stephens (2003) used to model the effect of recombination on haplotype diversity. We calculate the probability of observing a target sequence (conditional on specified values of transition and emission parameters) by assuming that the target is an imperfect mosaic of the other sequences in the sample allowing for insertion and deletion events. It is most straight forward to describe the model in terms of simulation. Consider a set of n source sequences. The target sequence is generated as follows:

- Choose the starting point of the novel sequence uniformly from all sites in the source sequences.
- With probability π_M the novel sequence starts in a match state. With probability I the novel sequence starts in an insert state.
- The novel sequence is constructed by sampling from a Markov chain that explores match, insert and delete states as in a typical pair-HMM.
- If the novel sequence is in a match state an emission matrix is used to sample the state conditional on the state of the source sequence at that position.
- If the novel sequence is in an insert state, the state is sampled from the stationary distribution of the emission matrix.
- At each step there is some probability of jumping, through recombination, to any other position on any other sequence (to either a match or insert state). As with initiation, the destination of the recombination event is chosen uniformly among all sites in the source sequences.
- At each step there is some probability of terminating.

Note that while the model lacks biological realism because of the ability to jump from any place to any other through recombination, this feature enables efficient inference through dynamic programming and importantly does not require sequences to be aligned prior to analysis, a property that is extremely important for a gene family as diverse as the *var* genes. For each sequence in turn, we calculate the likelihood of the model parameters. Parameter estimation is performed using a composite likelihood obtained by multiplying the likelihoods for each sequence in turn. We now given formal descriptions of the Viterbi, forward and backward algorithms used to calculate likelihoods.

Model Construction: Notation

Table 1 covers some of the common notation used from this point onwards. Important points to note from this notation include the following:

- The target sequence is represented as x, and the destination sequence as y.
- The emission probability is written as $e(x_i, y_j)$ where x_i is the amino acid at site i in sequence x and y_j is the amino acid at site j in sequence y. Similarly, the probability of observing a given amino acid emitted from an Insert state is written as $e(x_i)$.
- For clarity we write z as encompassing Match and Insert states.

Symbol	Meaning
n	Number of sequences in sample
x	Target sequence
y	Specific instance of destination sequence, so that $y \in \{1...h\}$
i	Index for position in target sequence x
j	Index for position in destination sequence y
k	Index for destination sequence so that $k \in \{1...n\}$
l	Length of destination sequence
m	Length of target sequence
w	All lengths of all sequences so that $w \in \{1...l_k\}$
h	Sequence set
a	Transition probability
q	State where a transition originates
r	State where a transition ends
e	Emission probability
z	Match and Insert states so that $z \in \{M; I\}$.
	Two other states, B and T represent Begin and Termination states.
$v_k^q(i,j)$	Example notation:
	v Algorithm identity;
	k Destination sequence identity;
	q State identity;
	i, j Positions along respective sequences.

Table S1. Notation used in the HMM equations. Thus includes an example notation format of the kind used in the following algorithm explanations.

Model Construction: Transition Matrix

The pair-HMM with recombination used here is described by the following transition matrix (which defines all transition probabilities of moving from state to state). Each row of the matrix must sum to one. The i, jth entry of the N by N transition matrix (where N is the number of states) is the probability of a transition from state i to state j.

For clarity, the matrix below denotes movements in a 3 sequence state where the subscript "x" represents the target sequence and "k" denotes any other destination sequence in the dataset.

	B	M_x	I_x	D_x	M_k	I_k	D_k	T
B	0	$\frac{\pi_M}{\|Y\|}$	$\frac{\pi_I}{\|Y\|}$	0	$\frac{\pi_M}{\|Y\|}$	$\frac{\pi_I}{\|Y\|}$	0	0
M_x	0	$1-2\delta-\rho-\tau$	δ	δ	$\frac{\rho}{\|Y\|}\pi_M$	$\frac{\rho}{\|Y\|}\pi_I$	0	τ
I_x	0	$1-\epsilon-\rho-\tau$	ϵ	0	$\frac{\rho}{\|Y\|}\pi_M$	$\frac{\rho}{\|Y\|}\pi_I$	0	τ
D_x	0	$1-\epsilon$	0	ϵ	0	0	0	0
M_k	0	$\frac{\rho}{\|Y\|}\pi_M$	$\frac{\rho}{\|Y\|}\pi_I$	0	$1-2\delta-\rho-\tau$	δ	δ	τ
I_k	0	$\frac{\rho}{\|Y\|}\pi_M$	$\frac{\rho}{\|Y\|}\pi_I$	0	$1-\epsilon-\rho-\tau$	ϵ	0	τ
D_k	0	0	0	0	$1-\epsilon$	0	ϵ	0
T	0	0	0	0	0	0	0	1

Here, δ is the probability of gap (indel) initiation, ϵ is the probability of gap (indel) extension, ρ is the probability of recombination, $\mid Y \mid$ is $\sum_{k=1}^{n} l_k$ where l_k = length of sequence k, π_M is the stationary probability of starting in match state, π_I is the stationary probability of starting in insert state, τ is the probability of termination, and n is the n number of destination sequences.

Viterbi algorithm

The Viterbi algorithm finds the most probable (maximum likelihood) path through the HMM given the sequence set h . By doing so it finds the best alignment, as it finds the path $\pi*$ that maximizes the joint probability $P(x|h, \pi, M)$. Following common dynamic programming techniques, the best alignment is found by keeping pointers during the recursion and tracing back, adding residues emitted by the HMM and the destination sequence identity to the alignment. While the Viterbi can produce a quantity which, when used with the full probability of the model, can indicate how correct the most probable path is, the main use of the Viterbi here arises from the optimal alignment pathway produced through the sequence set.

Initialization

v_k^M, v_k^I, v_k^D are $m+2$ by l_k+2 matrices.

$v_k^M(0,j) = v_k^M(i,0) = v_k^M(m+1,j) = v_k^M(i,l_k+1) = 0$

$v_k^I(0,j) = v_k^I(i,0) = v_k^I(m+1,j) = v_k^I(i,l_k+1) = 0$

$v_k^D(0,j) = v_k^D(i,0) = v_k^D(m+1,j) = v_k^D(i,l_k+1) = 0$

except for

$v_k^M(1,j) = \frac{\pi_M}{|Y|} e(x_1, y_j)$

and

$v_k^I(1,j) = \frac{\pi_I}{|Y|} e(x_1)$

Recurrence

For $1 = 1, ...m, j = 1, ..., l_k$ except $v_k^D(m, \cdot)$:

$$v_k^M(i,j) = e(x_i, y_j) \cdot \max \begin{cases} (1-2\delta-\rho-\tau) v_k^M(i-1,j-1) \\ (1-\epsilon-\rho-\tau) v_k^I(i-1,j-1) \\ (1-\epsilon) v_D^I(i-1,j-1) \\ \frac{\rho}{|Y|}\pi_M \max_{k,j,z} v_k^z(i-1,j) \end{cases}$$

$$v_k^M(i,j) = e(x_i, y_j) \cdot \max \begin{cases} (1-2\delta-\rho-\tau) v_k^M(i-1,j-1) \\ (1-\epsilon-\rho-\tau) v_k^I(i-1,j-1) \\ (1-\epsilon) v_D^I(i-1,j-1) \\ \frac{\rho}{|Y|}\pi_M \max_{k,j,z} v_k^z(i-1,j) \end{cases}$$

$$v_k^D(i,j) = \max \begin{cases} \delta v_k^M(i-1,j) \\ \epsilon_k^D(i-1,j) \end{cases}$$

Termination

$$v^E = \tau \max_{k,j,z} \{v_k^z(l,j)\} \tag{1}$$

Forward Algorithm

The forward probability is the probability that the HMM generates a particular sequence of observations.

$$P(x|h) = \sum_{\pi} P(x|\pi)P(\pi). \tag{2}$$

This is equivalent to the product of the probability of the target sequence given a path and the probability of that path, summed over all possible paths through the HMM. This has many uses, such as the derivation of posterior probabilities according to any alignment, and the calculation of a composite likelihood over all targets x in the sequence set h . This composite likelihood is used in maximum likelihood estimation.

Initialization

f_k^M, f_k^I, f_k^D are $m+2$ by l_k+2 matrices.

$f_k^M(0,j) = f_k^M(i,0) = f_k^M(m+1,j) = f_k^M(i,l_k+1) = 0$

$f_k^I(0,j) = f_k^I(i,0) = f_k^I(m+1,j) = f_k^I(i,l_k+1) = 0$

$f_k^D(0,j) = f_k^D(i,0) = f_k^D(m+1,j) = f_k^D(i,l_k+1) = 0$

except for

$f_k^M(1,j) = \frac{\pi_M}{|Y|} e(x_1, y_j)$

and

$f_k^I(1,j) = \frac{\pi_I}{|Y|} e(x_1)$

Recurrence

For $i = 1, \cdots, m$, $j = 1, \cdots, l_k$ except $v_k^D(m, \cdot)$:

$$
\begin{aligned}
f_k^M(i,j) =& e(x_i,y_j)[f_k^M(i-1,j-1)(1-2\delta-\rho-\tau) \\
&+ f_k^I(i-1,j-1)(1-\epsilon-\rho-\tau) \\
&+ f_k^D(i-1,j-1)(1-\epsilon) \\
&+ \frac{\rho}{\mid Y \mid}\pi_M \sum_{k=1}^{n}\sum_{j=1}^{w}\sum_{z} f_k^z(i-1,j)]
\end{aligned}
$$

$$
\begin{aligned}
f_k^I(i,j) =& e(x_i)[f_k^M(i-1,j)\delta \\
&+ f_k^I(i-1)\epsilon \\
&+ \frac{\rho}{\mid Y \mid}\pi_I \sum_{k=1}^{n}\sum_{j=1}^{w}\sum_{z} f_k^z(i-1,j)]
\end{aligned}
$$

$$
f_k^D(i,j) = f_k^M(i,j-1) + f_k^D(i,j-1)\epsilon
$$

Termination

$$
f = \tau \sum_{k=1}^{n}\sum_{j=1}^{w}\sum_{z} f_k^z(m,\cdot) \tag{3}
$$

Backward Algorithm

The backward algorithm is analogous to the forward algorithm but moving in the reverse direction through the algorithm. Thus $b_k^q(i,j)$ is the probability of the partial observed sequence from k,i,j to the end of the sequences, given state q at the point k,i,j and the model M .

The backward algorithm is used both in the analysis of posterior probabilities and in parameter estimation. Additionally, Equation 4 can be checked against Equation 3 to test for programming errors as their solutions are identical.

Initialization

b_k^M, b_k^I, b_k^D are $m+2$ by l_k+2 matrices.

$$b_k^M(0,j) = b_k^M(i,0) = b_k^M(m+1,j) = b_k^M(i,l_k+1) = 0$$

$$b_k^I(0,j) = b_k^I(i,0) = b_k^I(m+1,j) = b_k^I(i,l_k+1) = 0$$

$$b_k^D(0,j) = b_k^D(i,0) = b_k^D(m+1,j) = b_k^D(i,l_k+1) = 0$$

except for

$$b_k^M(m,l_k) = b_k^i(m,l_k) = \tau$$

Recurrence

For $i = m, ..., 1$, $j = l_k, ..., 1$ except $b^D(m,\cdot)$:

$$\begin{aligned} b_k^M(i,j) =& b_k^M(i+1,j+1)(1-2\delta-\rho-\tau)e(x_{i+1},y_{j+1}) \\ &+ b_k^I(i+1,j)\delta e(x_{i+1}) \\ &+ b_k^D(i,j+1)\delta \\ &+ \frac{\rho}{\mid Y \mid}\pi_M \sum_{k=1}^{n}\sum_{j=1}^{w} b_k^M(i+1,j)e(x_{i+1},y_{j+1}) \\ &+ \frac{\rho}{\mid Y \mid}\pi_I \sum_{k=1}^{n}\sum_{j=1}^{w} b_k^I(i+1,j)e(x_{i+1}) \end{aligned}$$

$$\begin{aligned} b_k^I(i,j) =& b_k^I(i+1,j)\epsilon e(x_{i+1}) \\ &+ b_k^M(i+1,j+1)(1-\epsilon-\rho-\tau)e(x_{i+1},j_{j+1}) \\ &+ \frac{\rho}{\mid Y \mid}\sum_{k=1}^{n}\sum_{j=1}^{w} \pi_M b_k^M(i+1,j)e(x_{i+1},y_{j+1}) + \pi_I b_k^I(i+1,j)e(x_{i+1}) \end{aligned}$$

$$b_k^D(i,j) = b_k^D(i,j+1)(1-\epsilon)e(x_{i+1},y_{j+1})$$

Termination

$$b = \sum_{k=1}^{n} \sum_{j=1}^{w} b_k^M(1,j) \frac{\pi_M}{| Y |} e(x_1, y_j) + b_k^I(1,j) \frac{\pi_I}{| Y |} e(x_1) \qquad (4)$$

Data analysis

Data analysis proceeds in two parts. Initially, with the recombination parameter set to zero, the transition and emission probabilities are estimated by EM using the Baum-Welch algorithm (note that it is the composite-likelihood is maximized). These parameters are then fixed and a likelihood surface is constructed for the recombination parameter. Once the MLE for the recombination parameter is found, the Viterbi path is constructed for each target sequence in turn. This path is used to provide the mosaic alignments summarized in the figures.

Algorithm performance

Simulation protocol

Our approach is to model the phenomenon of mosaicism observed in present day sequences rather than the process of gene-family evolution itself. To explore the behavior of the model fully, however, it is highly useful to be able to test the model upon datasets wherein factors such as phylogenies and parameters are already known, a situation that normally requires a model for *var* gene evolution. We do not attempt to fully model the complexity of the *var* sequences, however. Instead, different simulation approaches are used with an aim to capturing key features of *var* gene evolution and allowing us to test the robustness of the model. Two simulation approaches are used:

- The coalescent with recombination (Hudson 2002), along with a model of complex sequence evolution with site-specific substitution rates (Rambaut and Grassly 1997). Recombinant phylogenies were generated which represent data partitioned into differing phylogenetic histories. These were simulated using the coalescent with different crossover and gene conversion parameter values and assuming a neutral model with homologous recombination. Those partitioned phylogenies were then used to simulate amino acid sequences with recombinant histories, and the sequences were evolved using the BLOSUM62 substitution matrix and a site-specific substitution rate heterogeneity which was gamma-distributed with a parameter of 1 (Rambaut and Grassly 1997).

- Indel formation in sequence families without recombination. To simulate sequences that represent gene families containing highly related motifs, families of related sequences from a common ancestor sequence were simulated through a process of insertion, deletion and substitution of characters without recombination (Rambaut and Grassly 1997).

Although it would be useful to combine both features of recombination and indel formation, no program currently exists for this kind of joint simulation for amino acid datasets. The above protocol instead allows the investigation of particular features of *var* gene sequences.

For instance, the simulation of sequences which contain indels and sequence motifs in the presence of no recombination is useful because these features are a potential confounding factor for estimating recombination.

Program	Parameter	Value
Seq-Gen	Model of substitution	BLOSUM62
Seq-Gen	Site specific rate heterogeneity	γ-distributed, shape parameter 1
ms	r (Crossover rate)	0, 1, 10, 50
ms	g (Gene conversion rate)	1, 10, 50
ms	Average tract length of gene conversion	20
Rose	Model of substitution	PAM
Rose	Probability of insertion of certain length	0.00005
Rose	Probability of deletion of certain length	0.00005

Table S2. Values used in simulation programs

Another useful aspect of this simulation protocol is that it allows the recombination parameter used in our model to be related to the recombination parameter of the coalescent process using a calibration method described below. This calibration, performed on simulation results, is highly accurate allowing us to make comparisons between the ρ parameter and recombination parameters in coalescent models.

It is important to note that the coalescent is likely to be an inaccurate description of true *var* gene evolution. However, aspects of the coalescent represent several features of *var* gene family evolution because basic coalescent processes of coancestry and allelic recombination may represent *var* gene duplications and nonallelic recombination. It is also possible to model gene conversion through the coalescent (Wiuf and Hein 2000) a feature which is often highly important in the evolution of gene families (Ohta 1983).

Input parameters for these programs as listed in Table S2 were used to produce 60 simulated sequences with length of 150 sites.

Estimated values of transition probabilities

Parameter estimation was investigated with respect to recombination in terms of both gene conversion events and crossover events. Increases of the simulated crossover rate moved from $r = 1$ to $r = 50$, and increases of the simulated gene conversion rate moved from $g = 1$ to $g = 50$. A neutral dataset with no indel formation, crossover or gene conversion is also simulated ($r = 0$, $g = 0$), as well as a dataset with no recombination processes but with indel formations (indels). These simulated datasets can thus be summarized as follows:

- $r = 0$, $g = 0$: no crossover, conversion or indel formation processes
- indels: no recombination processes, high indel formation rate
- $r = 1$: low crossover rate
- $g = 1$: low conversion rate

- $r = 10$: moderate crossover rate
- $g = 10$: moderate conversion rate
- $r = 50$: high crossover rate
- $g = 50$: high conversion rate

The procedure for parameter estimation detailed above was followed for 10 simulated datasets in each of these categories. Baum Welch estimation of δ and ϵ with $\rho = 0$ usually led to parameters stabilizing within 5-10 iterations. The ρ parameter was then changed by 0.0001 per iteration in a grid search of the likelihood space for the maximum value. Results in the form of average values can be found in Table S3, and the distributions of these values can be seen in Figures S1, S2, and S3.

It can be seen (Figure S1) that the gap initiation parameter δ is highly sensitive to the existence of indels in a dataset, but is also sensitive to the presence of gene conversion within a dataset ($g = 1$, $g = 10$ and $g = 50$). However it is less sensitive to the presence of crossovers unless they occur at very high rates ($r = 50$). In contrast, the gap extension parameter ϵ shows no differences in sensitivity to crossover versus gene conversion events (Figure S2). In datasets with no indels, the ϵ parameter remains at a stable rate, except for instances where it is greatly increased, and within datasets with high recombination rates these events of high estimated ϵ appear to occur more frequently.

	Gap initiation δ	Gap extension ϵ	Recombination ρ	Composite Log Likelihood
$r = 0$, $g = 0$	0.000118	0.359627	0.000288	-2734.146
‘indels’	0.002071	0.816823	0.00176	-12069.547
$r = 1$	0.000113	0.335785	0.000344	-2675.372
$g = 1$	0.000371	0.391190	0.000402	-2634.446
$r = 10$	0.000120	0.419876	0.001408	-2826.724
$g = 10$	0.000471	0.506203	0.001416	-2971.568
$r = 50$	0.000223	0.484789	0.005425	-3433.202
$g = 50$	0.000989	0.630480	0.005553	-3740.350

Table S3: Average parameter estimation results for simulated data. See Figures S1, S1, and S3 for distributions of these estimated parameters.

Figure S3 shows the response of the recombination parameter ρ to the different simulated datasets. In datasets with no recombination or indel processes, a low level of recombination was estimated by the algorithm (note that some bias is expected, because rho must be positive). An overlap between estimates of ρ occurs between datasets with no recombination ($r = 0$) and datasets with low recombination ($r = 1$ and $g = 1$). However it can be seen that estimates of ρ respond in a roughly linear fashion to the presence of moderate to high recombination processes. It can be seen that estimates of ρ in the presence of either crossovers or gene conversion events are similar. Naively one would expect the detected amount of recombination in a dataset with gene conversion to be twice that of a dataset

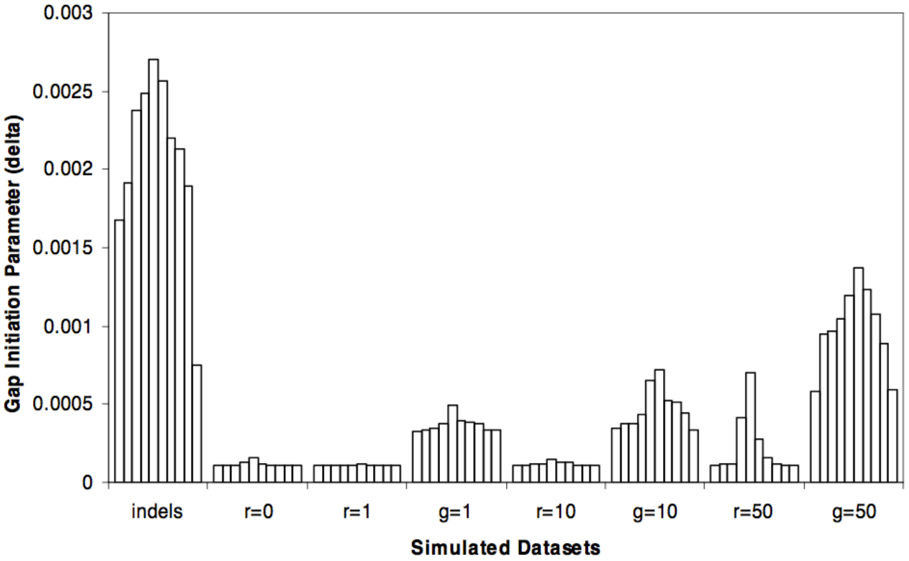

Figure S1: Simulation results of the parameter controlling gap initiation, δ. The δ parameter is estimated to be high in datasets with high numbers of indels (indels). δ values are increased in datasets containing gene conversion events ($g = 1$, $g = 10$ and $g = 50$), but only appear to be sensitive to crossover events when the rate of crossover is high ($r = 50$). Datasets with lower ($g = 1$ and $g = 10$) crossover rates or no recombination processes ($r = 0$) have very consistent values of δ. For clarity, results of individual simulations have been ordered so that the highest value is centered.

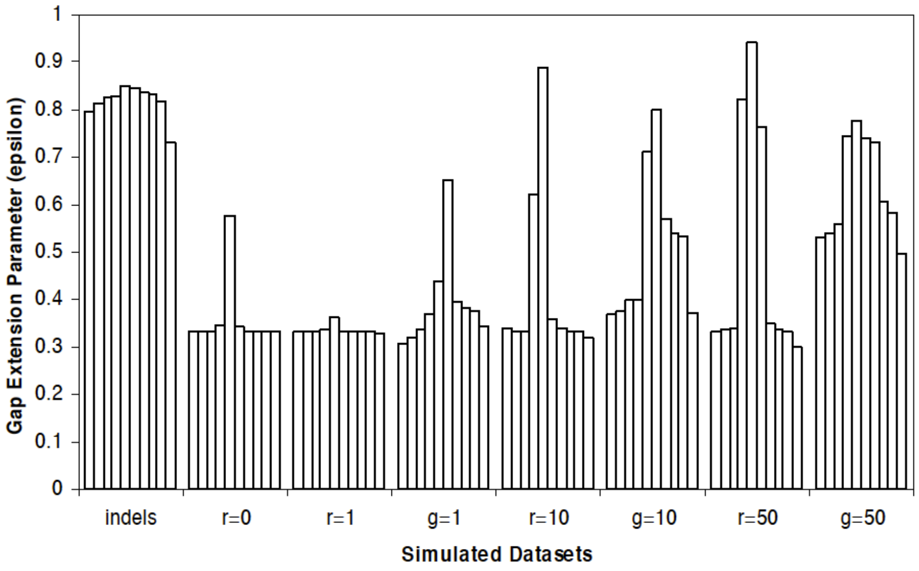

Figure S2: Simulation results of the parameter controlling gap extension, ϵ. Some datasets show elevated values of ϵ, especially those with higher recombination rates ($r = 10$, $g = 10$, $r = 50$ and $g = 50$), but again, the datasets containing true indels (indels) consistently estimate high values of ϵ. For clarity, results of individual simulations have been ordered so that the highest value is centered.

with crossover, as gene conversions are effectively double crossovers. Here, datasets with gene conversion increase the estimated value of ρ only slightly, but increase the value of δ more significantly. This indicates that although the model can detect large gene conversion events as recombination, small gene conversion events are detected as indels.

It can be seen that the recombination parameter is influenced by datasets with high numbers of indels, but no recombination (Figure S3). The reverse situation, the estimation of δ with a dataset with moderate to high amounts of recombination ($r = 50$ or $g = 50$) but no indels shows an increase in estimates of δ (Figure S1), especially in datasets with gene conversion events. Similarly, increasing amounts of recombination cause the ϵ parameter to increase in some datasets. In cases of high numbers of indels and no recombination, the algorithm is able to detect the former well. The relationships between the parameters δ and ρ are summarized in Table S4.

Relating ρ to coalescent recombination parameters

The true probability of recombination at a particular site in the dataset, r_{ij}, approximates to $\frac{\rho_{ij}}{\rho_{ij}+(n-1)}$ where ρ_{ij} is the estimated recombination probability and n is the number of sequences in the dataset. This itself can be approximated by $\frac{\rho_{ij}}{n}$ because of the very small value of ρ when divided by the total length of the sequence, $\mid Y \mid$. As a result,

$$\rho_{ij} \approx r_{ij} n \tag{5}$$

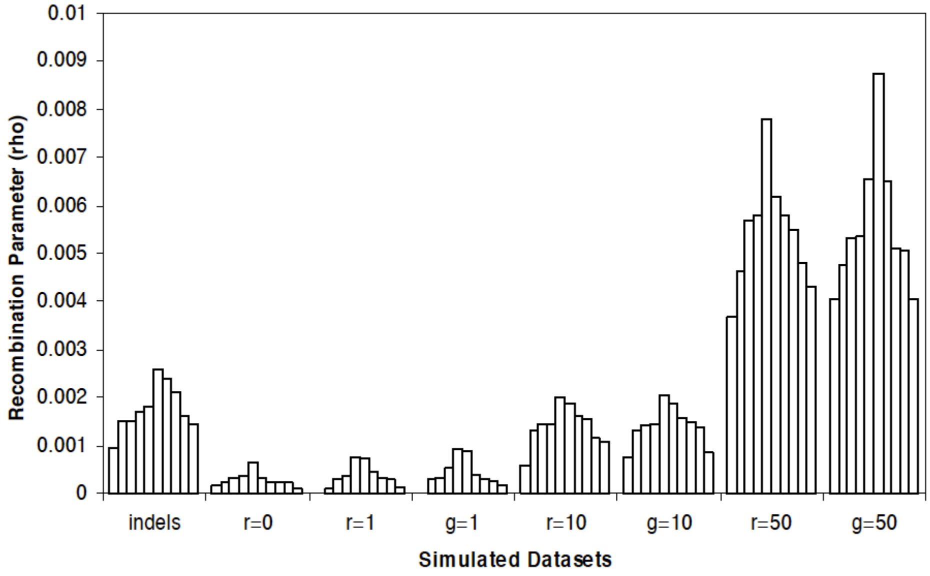

Figure S3: Simulation results of the parameter controlling recombination. The distributions of estimated values of ρ in the eight simulated datasets (as described in Table S2) show that the model has a slight bias towards overestimating values of ρ. The datasets '00' ($r = 0$, $g = 0$, no recombination) and '01' ($r = 0$, $g = 1$, low levels of recombination) have comparable values of ρ, however the datasets '10' and 'indels' also have similar values. However, values of ρ for the '50' dataset show considerably higher estimated values. For clarity, results of individual simulations have been ordered so that the highest value is centered.

	δ =high	δ =low
ρ =high	unknown	upward bias in δ<br>accurate ρ
ρ =low	accurate δ<br>upward bias in ρ	accurate δ<br>accurate ρ

Table S4: The relationship between the estimation of the parameters δ and ρ. Although there is no program currently available that allows the simulation of the situation wherein both indel rates and recombination rates are high in amino acid data, simulated data with high ρ values and low δ values have estimated parameters which are accurate for ρ and slightly overestimated for δ. In simulated data with low ρ values, estimated parameters are accurate when δ is also low, and show a bias towards overestimation of ρ when δ is high.

and we can assess the accuracy of the simulations according to a coalescent framework by finding the value of $\frac{\rho}{l}\frac{1}{n}$, results shown in Table S5.

ρ/l	n	true r_{ij}	mean estimated ρ (crossover)	mean estimated ρ (gene conversion)
1/150	60	0.000111	0.000344	0.000402
10/150	60	0.000111	0.001408	0.001416
50/150	60	0.005556	0.005425	0.005553

Table S5: Comparison of true and estimated values of ρ. Estimated values of ρ show noteable accuracy compared to the true value as calculated by $\frac{\rho}{l}\frac{1}{n}$.

Testing for the presence of recombination

A likelihood ratio test can be applied to test the hypothesis that recombination exists in the datasets using the quantity $2\ (L_{\hat{\rho}} - L_{\rho=0})$ where $L_{\hat{\rho}}$ is the log likelihood at the estimated value of ρ and L_ρ =0 the log likelihood with no recombination. It is often assumed that this approximates to a χ^2 distribution. However there is no reason to make that assumption here. To test our results formally, a simulation would be necessary to find the expected null distribution. Such a test would provide a powerful test of recombination, but we do not undertake it, namely because one of the *ab initio* assumptions of our model is that there is recombination present in the data. Instead, we can examine the results of the likelihood ratio values from the simulated datasets (Table S6), and this illustrates how the distribution of these values responds to the amount of recombination in the datasets. There is a marked response in the likelihoods to the amount of recombination present in the data.

Dataset	Range of $2\ (L_{\hat{\rho}} - L_{\rho=0})$
$r = 0$, $g = 0$	4.26 - 48.42
'indels'	78.40 - 278.00
r=1	8.70 - 239.24
g=1	2.45 - 111.55
r=10	197.84 - 924.30
g=10	141.16 - 521.36
r=50	1605.90 - 3902.50
g=50	1145.60 - 2611.16

Table S6: Likelihood ratio test results for simulated data. The range of values is considerable but is considerably higher in datasets with high recombination rates. There is an overlap of values for datasets with $r = 0$ and $r = 1$ or $g = 1$.

Estimated values of emission probabilities

Simulated values of the 20x20 pair emission matrix, eij and the 1x20 gap matrix, ei, were investigated using heat matrices. The average values of the ten simulated datasets for $r = 0$, $g = 0$, indels, $r = 1$, $r = 10$, and $r = 50$ can be seen in Figure S4. Results for $g = 1$,

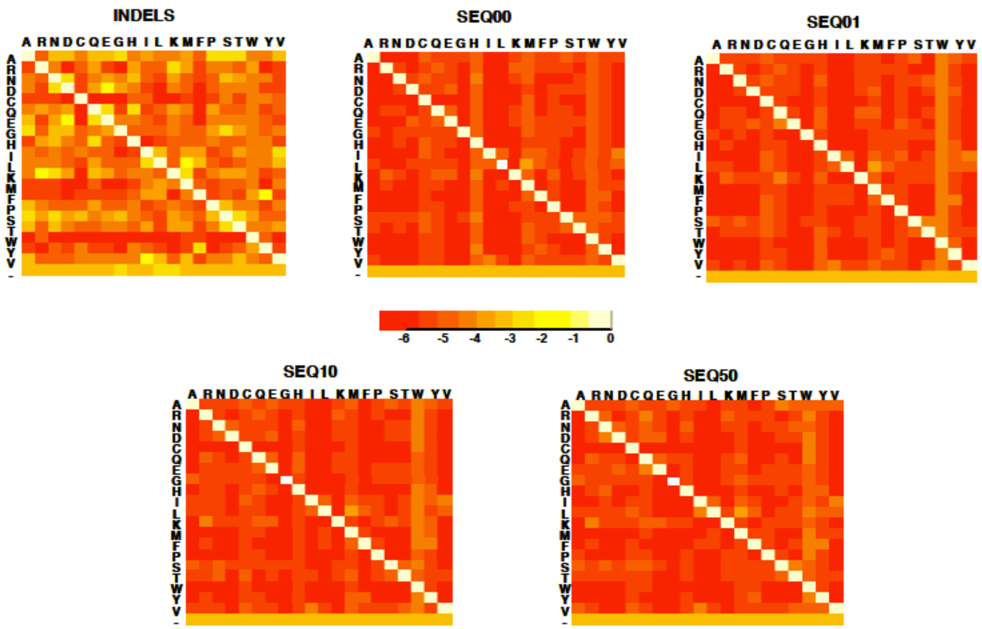

Figure S4: Simulation results of the e_{ij} parameters. Log scores of the substitution matrices estimated using EM methods were visualized using a color gradient. Red entries denote low probabilities, orange medium, and white entries denote high probabilities. Note how the use of simulated datasets with indels affects the emission probability scores considerably. INDELS refers to the indels dataset; SEQ00 to $r = 0$, $g = 0$, SEQ01 to $r = 1$, SEQ10 to $r = 10$, and SEQ50 to $r = 50$.

$g = 10$ and $g = 50$ are highly similar and are not shown. After simulation on datasets with no indels, the model estimated substitution matrices which penalize mismatched amino acid pairs heavily. This was regardless of recombination value. In comparison, and as expected, the indel dataset displays a much more heterogenous substitution matrix. All simulated values of the 1x20 gap matrix stabilized around 0.05.

Speed and underflow

The multiplication of many probabilities in HMMs leads quickly to numerical stability problems. Transforming the models equations into log space avoids potential underflow errors since the log of a product is the sum of the logs. All calculations were carried out in log space as detailed above. However, because summing of probabilities also occurs (for instance in the forward algorithm), log space calculations quickly become quite complex.

Both the complexity and the memory usage of the model grow as $O(l^2k)$ where l is the length of sequences and k the number of sequences. As a comparison, the Needleman-Wunsch algorithm takes $O(l^2)$ time and memory (Durbin et al. 1998).

References

Durbin R, Eddy SR, Krogh A, Michison G (1998) Biological sequence analysis: probabilistic models of proteins and nucleic acids: Cambridge University Press.

Hudson RR (2002) Generating samples under a Wright-Fisher neutral model of genetic variation. Bioinformatics, 18:337-338.

Li N, Stephens M (2003) Modeling linkage disequilibrium and identifying recombination hotspots using single nucleotide polymorphism data. Genetics 165:2213-2223.

Ohta T (1983) On the evolution of multigene families. Theoretical Population Biology, 23:216-240.

Rambaut A and NC Grassly (1997) Seq-Gen: an application for the Monte-Carlo simulation of DNA sequence evolution along phylogenetic trees. Computational Applied Biosciences, 13:23-238.

Stoye J, Evers D, Meyer F (1998) Rose: generating sequence families. Bioinformatics, 14:157-163

Wiuf C, Hein J (2000) The coalescent with gene conversion. Genetics, 155:451-462
