# Supplementary material for: Hypervariable antigen genes in malaria have ancient roots
Source: BMC Evol Biol. 2013 May 31;13:110. doi: 10.1186/1471-2148-13-110 (PMC3680017; doi:10.1186/1471-2148-13-110)
Supplement: Additional file 2 — Analysis and Results of Core Motifs and Conserved Peptides. An MS Word document describing and summarizing the analysis of core motifs and conserved peptides between P. reichenowi and P. falciparum. [file 1471-2148-13-110-S2.docx]

***Analysis and Results of Core Motifs and Conserved Peptides***

In further examination of the *P. reichenowi* and *P. falciparum* DBLα domain homology blocks, we revealed multiple regions of high sequence homology, classified here in two groups: core motifs and conserved peptides. The core motifs are defined as regions of 100% sequence identity that are found in at least one *P. reichenowi* gene matching two or more from *P. falciparum* (unique pairs of matches are considered conserved peptides, details below) and correspond to one of the five known degenerate motifs [[1](#_ENREF_1)] that characterize the DBLα domains. These domains, listed starting from the N-terminus side are HB4, HB3, HB5, HB2, and HB1 [[1](#_ENREF_1)]. We recovered 18 sequences corresponding to the conserved motifs, between 10 and 29 residues, in 36 different *P. reichenowi* *var* genes and shared with 85 of those from 3D7 and HB3. The HB2 motifs are the most frequently represented in this group, corresponding to 11 of the 18 different motifs, and were found in 26 *P. reichenowi* and 63 *P. falciparum* paralogs total, with a mid-sized motif (19 residues) found in 16 out of 49 standard (non-*csa*) *P.reichenowi* PFEMP1 sequences. The HB3 motifs are found in two different forms, in 12 *P. reichenowi* and 54 *P. falciparum* domains, and HB5 in four forms in 9 *P. reichenowi* genes and 19 from the *P. falciparum* sets. The HB4 had only one identical form matching two *P. falciparum* (one each in 3D7 and HB3). Notably, HB1 motifs were not present in these results. Although one match was found for this motif, it is not counted among these results in this group as it was only found in a unique pair, and it was between the *P. reichenowi* and HB3 *var1csa* genes, a locus which is already known to have a relatively recombination rate and high degree of conservation, quite distinct from the standard PfEMP1s (these results are summarized in Table S7).

The *var* genes are classified as upsaA, upsB, or upsC based on conserved upstream sequences. A combination of all the results of the *P. falciparum* matches from all the motifs shows a total of 555 hits, with individual genes represented between 1 and 14 times. Of these 555 hits, 13 were from uspA genes, 368 were from upsB, and 164 from upsC. Because there are a total of 18 upsA, 55 upsB, and 21 upsC genes in the analysis, this indicates that upsB and upsC genes show greater levels of conservation, with upsA genes more divergent [[2](#_ENREF_2)].

In addition to these core motifs, we also found 53 regions that define conserved peptides, determined by pairwise matches of high identity/similarity between *P. reichenowi* and *P. falciparum* DBLα domains. These peptides range between 24 and 140 residues in length, and between 70% and 100% identity (identical amino acids) and 80% to 100% similarity (amino acids with the same or similar functional characteristics). The conserved regions are drawn from 48 non-redundant matches, with the five additional peptides each drawn from a smaller portion of a conserved peptide with even higher identity. While many *P. reichenowi* loci revealed none of this highly conserved sequence, some loci were represented in multiple matches, the most frequent being Preich_4 with 6 non-redundant regions plus one additional sub-region of high *P. reichenowi*/*P. falciparum* homology, Preich_11 with four peptides and one additional sub-region of high identity, and Preich_7.2, and Preich_84, both with four matches

1. Rask TS, Hansen DA, Theander TG, Gorm Pedersen A, Lavstsen T: ***Plasmodium falciparum* Erythrocyte Membrane Protein 1 Diversity in Seven Genomes: Divide and Conquer**. *PLoS Comput Biol* 2010, **6**(9):e1000933.

2. Lavstsen T, Salanti A, Jensen ATR, Theander TG, Copenhagen D: **Sub-grouping of Plasmodium falciparum 3D7 var genes based on sequence analysis of coding and non-coding regions**. *Malaria Journal* 2003, **2**:27.

| **Sequence** | **Matches** | **Motif** | **Length** | **No. Matches: *P. reichenowi*** | **No. Matches: *P. falciparum*** |
| --- | --- | --- | --- | --- | --- |
| VFEVWLGNQQEAFKKQK | 2 | HB1 | 17 | 1 | 1 |
| FDYVPQYLRWF | 87 | HB2 | 11 | 24 | 63 |
| FDYVPQYLRWFEEW | 78 | HB2 | 14 | 19 | 59 |
| FDYVPQYLRWFEEWAE | 75 | HB2 | 16 | 18 | 57 |
| DYVPQYLRWFEEWAEDFCR | 69 | HB2 | 19 | 16 | 53 |
| FDYVPQYLRWFEEWAEDFC | 70 | HB2 | 19 | 16 | 54 |
| FDYVPQYLRWFEEWAEDFCR | 67 | HB2 | 20 | 14 | 53 |
| PTYFDYVPQYLRWFEEWAEDFCR | 63 | HB2 | 23 | 11 | 52 |
| VPTYFDYVPQYLRWFEEWAEDFCRK | 40 | HB2 | 25 | 7 | 33 |
| DQVPTYFDYVPQYLRWFEEWAEDFCRK | 12 | HB2 | 27 | 3 | 9 |
| PTYFDYVPQYLRWFEEWAEDFCRKKKK | 23 | HB2 | 27 | 2 | 21 |
| PTYFDYVPQYLRWFEEWAEDFCRLRKHKL | 8 | HB2 | 29 | 2 | 6 |
| LARSFADIGDIVRG | 66 | HB3 | 14 | 12 | 54 |
| CTVLARSFADIGDIVRGRDLY | 5 | HB3 | 21 | 1 | 4 |
| APYRRRHICDYNLHHINENN | 3 | HB4 | 20 | 1 | 2 |
| WWTANRETVW | 16 | HB5 | 10 | 8 | 8 |
| KLREDWWTANR | 20 | HB5 | 11 | 5 | 15 |
| LREDWWTANRETVW | 15 | HB5 | 14 | 8 | 7 |
| KLREDWWTANRETVW | 8 | HB5 | 15 | 4 | 4 |

Table S7: Summary of core motifs

| *P. falciparum* | *P. reichenowi* | Identical Aas | Length | Percent Identity | Similar AAs | Percent Similarity |
| --- | --- | --- | --- | --- | --- | --- |
| HB3var16 | Preich_24 | 48 | 60 | 80 | 56 | 93 |
| HB3var16 | Preich_126 | 61 | 84 | 73 | 75 | 89 |
| HB3var16 | Preich_102 | 51 | 73 | 70 | 62 | 85 |
| HB3var17 | Preich_7.2 | 101 | 138 | 73 | 114 | 83 |
| HB3var18 | Preich_9 | 63 | 82 | 77 | 74 | 90 |
| HB3var18 | Preich_7.2 | 105 | 139 | 76 | 116 | 83 |
| HB3var21 | Preich_7.2 | 103 | 136 | 76 | 118 | 87 |
| HB3var22 | Preich_84 | 72 | 95 | 76 | 79 | 83 |
| HB3var24 | Preich_40 | 42 | 48 | 88 | 47 | 98 |
| HB3var25 | Preich_17 | 33 | 40 | 83 | 36 | 90 |
| HB3var27 | Preich_4 | 37 | 39 | 95 | 39 | 100 |
| HB3var27 | Preich_61 | 35 | 35 | 100 | x | x |
| HB3var2 | Preich_5.2 | 44 | 50 | 88 | 48 | 96 |
| HB3var2 | Preich_8.2 | 47 | 59 | 80 | 55 | 93 |
| HB3var34 | Preich_5 | 76 | 83 | 81 | 73 | 88 |
| HB3var34* | Preich_5* | 47 | 55 | 85 | 51 | 93 |
| HB3var34** | Preich_5** | 27 | 27 | 100 | x | x |
| HB3var36 | Preich_26 | 37 | 47 | 79 | 42 | 89 |
| HB3var36 | Preich_4 | 102 | 142 | 72 | 121 | 85 |
| HB3var36* | Preich_4* | 81 | 101 | 80 | 92 | 91 |
| HB3var6 | Preich_8.2 | 30 | 36 | 83 | 36 | 100 |
| HB3var7 | Preich_1 | 26 | 29 | 90 | 26 | 90 |
| HB3var8 | Preich_46 | 79 | 100 | 79 | 85 | 85 |
| HB3var8_2 | Preich_46 | 24 | 29 | 83 | 27 | 93 |
| MAL7P1.187 | Preich_7 | 31 | 36 | 86 | 33 | 92 |
| MAL7P1.56 | Preich_4 | 45 | 63 | 71 | 51 | 81 |
| MAL7P1.56* | Preich_4* | 36 | 40 | 90 | 38 | 95 |
| PF07_0050 | Preich_11 | 70 | 99 | 71 | 82 | 83 |
| PF07_0050 | Preich_7.2 | 39 | 46 | 85 | 43 | 93 |
| PF07_0050* | Preich_11* | 22 | 29 | 76 | 26 | 90 |
| PF070051 | Preich_4 | 37 | 46 | 80 | 40 | 87 |
| PF080103 | Preich_133 | 115 | 145 | 79 | 126 | 87 |
| PF080103* | Preich_4.2* | 32 | 35 | 91 | 35 | 100 |
| PF080103 | Preich_4.2 | 65 | 87 | 75 | 77 | 89 |
| PF080140 | Preich_11 | 23 | 29 | 79 | 26 | 90 |
| PF100406 | Preich_61 | 28 | 34 | 82 | 30 | 88 |
| PF110521 | Preich_11 | 23 | 30 | 77 | 29 | 97 |
| PF140773_truncated | Preich_5 | 29 | 37 | 78 | 35 | 95 |
| PF140773_truncated | Preich_61 | 53 | 67 | 79 | 58 | 87 |
| PFB1055c | Preich_25 | 49 | 59 | 83 | 53 | 90 |
| PFB1055c | Preich_84 | 31 | 37 | 84 | 34 | 92 |
| PFC0005w | Preich_32 | 57 | 73 | 78 | 61 | 84 |
| PFC0005w | Preich_46 | 72 | 101 | 71 | 81 | 80 |
| PFC0005w | Preich_11 | 21 | 30 | 70 | 26 | 87 |
| PFC0005w | Preich_84 | 37 | 41 | 90 | 41 | 100 |
| PFC1120c | Preich_84 | 43 | 58 | 74 | 50 | 86 |
| PFD1245c | Preich_9 | 47 | 56 | 84 | 52 | 93 |
| PFE1640w_truncated | Preich_8.2 | 48 | 58 | 83 | 52 | 90 |
| PFL0005w | Preich_5 | 28 | 37 | 76 | 34 | 92 |
| PFL1955w | Preich_40 | 38 | 43 | 88 | 41 | 95 |
| PFL1970w | Preich_78 | 67 | 92 | 73 | 74 | 80 |
| PFL2665c | Preich_129 | 97 | 139 | 70 | 117 | 84 |
| PFL2665c | Preich_4 | 21 | 24 | 88 | 24 | 100 |

Table S8: Summary of conserved peptide matches. Stars (*) indicate sub sections of higher homology
