# Supplementary material for: Hypervariable antigen genes in malaria have ancient roots
Source: BMC Evol Biol. 2013 May 31;13:110. doi: 10.1186/1471-2148-13-110 (PMC3680017; doi:10.1186/1471-2148-13-110)
Supplement: Additional file 4 — Alignments of conserved peptides. An MS Word document showing all alignments between P. reichenowi and P. falciparum for conserved peptides. [file 1471-2148-13-110-S4.docx]

Alignments of conserved peptides.

*P. reichenowi* sequences are on top, *P. falciparum* below.

Preich_001 HB3var7

YRRLHLCDQHLEHINSDKITTHNLLADVC

YRRLHLCDQHLEHI DKIT HNLLADVC

YRRLHLCDQHLEHIKRDKITRHNLLADVC

Preich_004.2 PF080103

AKTQYNDPEGNFYQLREDWWALNRKDVWKALTCDAPAGAQYFRLTCSDGNSSAHNRCTCVDGDPPTFFDYVPQYLRWFEEWAEDFCR

AK +YNDP+G+F+QLREDWWALNR+ VW A+TC+A G +YFR TCS G S AHN+CTC++GDPPT+FDYVPQYLRWFEEWAEDFCR

AKARYNDPKGDFFQLREDWWALNREKVWSAITCNA-QGNKYFRPTCSGGESIAHNKCTCINGDPPTYFDYVPQYLRWFEEWAEDFCR

Preich_004.2 PF080103*

AHNRCTCVDGDPPTFFDYVPQYLRWFEEWAEDFCR

AHN+CTC++GDPPT+FDYVPQYLRWFEEWAEDFCR

AHNKCTCINGDPPTYFDYVPQYLRWFEEWAEDFCR

Preich_004 MAL7P1.56

EKKDRYNGDKENFYQLREDWWTANRHTVWEAMTCNEDLKNSSYFRATCSDGKNQYQAHEKCRC

E + RYN NFYQLREDWWTANR T+WEAMTC+EDLKNSSYFR TCSD + QA++KCRC

EAQTRYNDATGNFYQLREDWWTANRATIWEAMTCSEDLKNSSYFRQTCSDERGGAQANDKCRC

Preich_004 MAL7P1.56*

NFYQLREDWWTANRHTVWEAMTCNEDLKNSSYFRATCSD

NFYQLREDWWTANR T+WEAMTC+EDLKNSSYFR TCSD

NFYQLREDWWTANRATIWEAMTCSEDLKNSSYFRQTCSD

Preich_004 PFL2665c

NFYQLREDWWTANRHTVWEAMTCN

NF++LREDWWTANRHTVWEAMTC+

NFFKLREDWWTANRHTVWEAMTCH

Preich_004 HB3var36

AMTCNEDLKNSSYFRATCSDGKNQYQAHEKCRCEKKRGANADQVPTYFDYVPQYLRWFEEWAEDFCRKRKKKLENLDKQCRGEYEREKRYCSRNGYDCEETVRARGKLRMGKGCISCLYACNPYIDWINNQKEQFLKQRNKY

AMTC+++L +SYF ATC DG ++ QA +KCRC+KK DQVPTYFDYVPQYLRWFEEWAE+FCRK+KKKLE L++QCRG+Y+ RYCSRNG+DCE+TV ARGK+RMGKGC C +ACNPYIDWINNQKEQF KQ+NKY

AMTCSDELTGASYFHATCIDGNSKSQAKDKCRCQKKDNKPNDQVPTYFDYVPQYLRWFEEWAEEFCRKKKKKLEKLEQQCRGKYQDADRYCSRNGFDCEKTVNARGKVRMGKGCTDCFFACNPYIDWINNQKEQFDKQKNKY

Preich_004 HB3var36*

DQVPTYFDYVPQYLRWFEEWAEDFCRKRKKKLENLDKQCRGEYEREKRYCSRNGYDCEETVRARGKLRMGKGCISCLYACNPYIDWINNQKEQFLKQRNKY

DQVPTYFDYVPQYLRWFEEWAE+FCRK+KKKLE L++QCRG+Y+ RYCSRNG+DCE+TV ARGK+RMGKGC C +ACNPYIDWINNQKEQF KQ+NKY

DQVPTYFDYVPQYLRWFEEWAEEFCRKKKKKLEKLEQQCRGKYQDADRYCSRNGFDCEKTVNARGKVRMGKGCTDCFFACNPYIDWINNQKEQFDKQKNKY

Preich_004 HB3var27

VPTYFDYVPQYLRWFEEWAEDFCRKRKKKLENLDKQCRG

VPTYFDYVPQYLRWFEEWAEDFCRK+KKKLENL+KQCRG

VPTYFDYVPQYLRWFEEWAEDFCRKKKKKLENLEKQCRG

Preich_004 PF070051

GAECANNRIEGNIKNSKNKDFGACAPYRRLSLCNKNFQNMNIKDSS

GAECANNRI+GN KNS KDFGACAP+RRL+LCNKNF NMN DSS

GAECANNRIQGNNKNSHYKDFGACAPFRRLNLCNKNFPNMNSNDSS

Preich_005.2 HB3var27

APYRRRHICDYNLHHINENNIKNTHDLLGNVLVMAKSEGESIVKSHEYTG

APYRRRHICDYNLHHINENNI+NTHDLLGNVLVMAKSEG SIV SH+++G

APYRRRHICDYNLHHINENNIRNTHDLLGNVLVMAKSEGASIVNSHKHSG

Preich_005 PF140773_truncated

RRLFLCDQNLEHIKPDKITNTHNLLVDVLLAAKYEGQ

RRL +CD+NLE IKPD+IT+THNLLVDVLLAAK+EG+

RRLHICDRNLELIKPDQITSTHNLLVDVLLAAKHEGK

Preich_005 PFL0005w

RRLFLCDQNLEHIKPDKITNTHNLLVDVLLAAKYEGQ

RRL++CD+NLEHI+P KIT THNLL+DV LAA+YEGQ

RRLYMCDRNLEHIEPTKIT-THNLLLDVCLAAQYEGQ

Preich_005 HB3var34*

GACAPLRRLFLCDQNLEHIKPDKITNTHNLLVDVLLAAKYEGQSIQKEYTQKGGDYKSGLCTALARSFADIGDIIRGKDLFIG

GACAPLRRL LCD+NLE IK +KI T NLLVDVLLAAKYEG+SI+ Y+QK DYKSGLCTALARSFADIGDIIRGKDLF+G

GACAPLRRLHLCDRNLEEIKYNKINQTDNLLVDVLLAAKYEGESIRNAYSQKSNDYKSGLCTALARSFADIGDIIRGKDLFLG

Preich_005 HB3var34**

NLLVDVLLAAKYEGQSIQKEYTQKGGDYKSGLCTALARSFADIGDIIRGKDLFIG

NLLVDVLLAAKYEG+SI+ Y+QK DYKSGLCTALARSFADIGDIIRGKDLF+G

NLLVDVLLAAKYEGESIRNAYSQKSNDYKSGLCTALARSFADIGDIIRGKDLFLG

Preich_005 HB3var34***

DYKSGLCTALARSFADIGDIIRGKDLF

DYKSGLCTALARSFADIGDIIRGKDLF

DYKSGLCTALARSFADIGDIIRGKDLF

Preich_007.2 HB3var21

PCGNRSKVRFSDKYGGQCTDTKIHGNDPKNGGACAPLRRLFLCDHHLSHMNHEKIDTTHNLLLEVSLAAKYEGKSIIENYPQDRNNKEGICIALARSFADIGDIIRGKDLFRGYNEKDKVQKEKLEKNLKDIFAKI

PCGNR VRFSDKYGGQCTD+KI GN+ +NGGACAP RRLFLCD +LS+M KID THNLLLEVSLAA++EG SIIENY + N+EGICIALARSFADIGDIIRGKDLF GYNEKDK +K++L+ +LK+IFAKI

PCGNRPDVRFSDKYGGQCTDSKIKGNNTENGGACAPFRRLFLCDQNLSYMKENKIDNTHNLLLEVSLAAQHEGDSIIENYIKRHENREGICIALARSFADIGDIIRGKDLFIGYNEKDKEEKKQLQDSLKNIFAKI

Preich_007.2 HB3var18

PCGNRSKVRFSDKYGGQCTDTKIHGNDPKNGGACAPLRRLFLCDHHLSHMNHEKIDTTHNLLLEVSLAAKYEGKSIIENYPQDRN---NKEGICIALARSFADIGDIIRGKDLFRGYNEKDKVQKEKLEKNLKDIFAKI

PC NR VRFSD YGGQCTD+KI GND NGGACAPLRRLFLCDHHLSHM KI+ HNLLLEVSLAAKYEG+SI+ N+P D+N NK GIC +LARSFADIGDIIRGKDLF GYNEKD+ +KEK++KNLK IF KI

PCKNRPNVRFSDIYGGQCTDSKIRGNDTNNGGACAPLRRLFLCDHHLSHMEEHKINDIHNLLLEVSLAAKYEGESIVNNHP-DKNSNGNKSGICTSLARSFADIGDIIRGKDLFIGYNEKDRKEKEKVQKNLKKIFRKI

Preich_007.2 HB3var17

PCGNRSKVRFSDKYGGQCTDTKIHGNDPKNGGACAPLRRLFLCDHHLSHMNHEKIDTTHNLLLEVSLAAKYEGKSIIENYPQDR--NNKEGICIALARSFADIGDIIRGKDLFRGYNEKDKVQKEKLEKNLKDIFAKI

PC NRS +RFSDKYGGQCTDTKIHGND GGACAP RRLFLCDHHLSHMN K +TT NLLLEV AAKYEG+S++EN+ + + NN IC ALARSFADIGDIIRGKDLF GYNEKD+ +K++L+ NLKDIF KI

PCANRSNIRFSDKYGGQCTDTKIHGNDEGPGGACAPFRRLFLCDHHLSHMNAGKTNTTDNLLLEVCYAAKYEGESLVENHEEYKKTNNDSNICTALARSFADIGDIIRGKDLFIGYNEKDREEKKQLQDNLKDIFKKI

Preich_007.2 PF07_0050

AAKYEGKSIIENYPQDRNNKEGICIALARSFADIGDIIRGKDLFRG

AAK+EG+SII+NYPQDRNN E IC ALARSFADIGDIIRGKDL+RG

AAKHEGESIIKNYPQDRNNNEVICTALARSFADIGDIIRGKDLYRG

Preich_007 MAL7P1.187

IFDKIKKSDTKLTSLKDEEIREYWWEANRETVWKAI

IF+ IKKSDTKLT L DE+IREYWWEANRETVWKAI

IFENIKKSDTKLTKLNDEQIREYWWEANRETVWKAI

Preich_008.2 HB3var6

TNLDYVPQFLRWFDEWAEDFCRIRKYKLEKVKEACR

TNLDYVPQFLRWFDEWAE+FCR+R++KL+K+KEACR

TNLDYVPQFLRWFDEWAEEFCRVREHKLKKIKEACR

Preich_008.2 HB3var2

NDSEKLYCSHNGYDCTKTIRNENILSDDPKCTGCLVKCSLYDSWLRNQRNEFEKQKEKY

"+DS+KLYCSHNGYDC +TIRNE+ILSD+PKCTGC VKC +Y+ WLRNQRNEFEKQK+KY"

DDSKKLYCSHNGYDCIETIRNEDILSDNPKCTGCSVKCKVYELWLRNQRNEFEKQKKKY

Preich_008.2 PFE1640w_truncated

DSEKLYCSHNGYDCTKTIRNENILSDDPKCTGCLVKCSLYDSWLRNQRNEFEKQKEKY

DSEKLYC HNGYDCTKTI+NENIL DDPKCT CL+KCSLY+ WL NQ+ EFEKQKEKY

DSEKLYCRHNGYDCTKTIKNENILFDDPKCTDCLIKCSLYEIWLDNQQKEFEKQKEKY

Preich_009 HB3var18

NANRKDIWKAMTCQAHDSDKYFREKDTEGRECTVKKCKCIDGDPPTNLDYVPQYLRWFEEWAEDFCRKRKHKLENAKNKCRG

NANRK++WKA+TC+A+D DKYFREK++ G CTV KCKC+DGDPPTNLDYVPQYLRWF+EW E+FCRKRK +LENAK KCRG

NANRKEVWKAITCKANDDDKYFREKNSNGNTCTVNKCKCVDGDPPTNLDYVPQYLRWFDEWGEEFCRKRKKQLENAKKKCRG

Preich_009 PFD1245c

EERYCSGNGYDCTQTVRAEDIYSMENNCHKCFFACNPFVKWLDKQKEEFNKQKEKY

+ERYCSGNGYDCTQTVRA++ YSMENNCHKCFFACNPFVKWL QK EF+KQK+KY

QERYCSGNGYDCTQTVRAQEEYSMENNCHKCFFACNPFVKWLGNQKLEFDKQKKKY

Preich_011 PF07_0050

NYYQLREDWWNANRKDVWKAITCDAPKDAYYTKKGLDGNIKDYALGQCRCITGDPPTNLDYVPQYLRWFEEWAEDFCRKRKKQLKDAIKICRGDDESGK

N++QLREDWWNANR D+WKA+TC AP DA YTKKG +I + GQCRC +GDPPTN+DYVPQYLRWFEEWAE+FCRKRKK+L++A K CR DES K

NFFQLREDWWNANRNDIWKALTCHAPPDAQYTKKGPHNHITESNKGQCRCFSGDPPTNMDYVPQYLRWFEEWAEEFCRKRKKKLQNAQKFCR--DESSK

Preich_011 PF110521

PNYYQLREDWWNANRKDVWKAITCDAPKDA

PNYY+LR+DWWNANR+DVWKA+TC+AP +A

PNYYKLRDDWWNANREDVWKALTCNAPYEA

Preich_011 PF07_0050

NYYQLREDWWNANRKDVWKAITCDAPKDA

N++QLREDWWNANR D+WKA+TC AP DA

NFFQLREDWWNANRNDIWKALTCHAPPDA

Preich_011 PF080140

NYYQLREDWWNANRKDVWKAITCDAPKDA

NY++LREDWW ANR VWKAITC+APKDA

NYFKLREDWWTANRDQVWKAITCNAPKDA

Preich_011 PFC0005w

PNYYQLREDWWNANRKDVWKAITCDAPKDA

P+++QLREDWW ANR+ VWKAI C AP+DA

PDFFQLREDWWTANRETVWKAIRCSAPRDA

Preich_017 HB3var25

DRYNGDEKKNFFKLREDWWTANRETVWKAITCEANGTYFR

+RY GDE NFFKLREDWWT NRETVWKAITC+A+GTYFR

ERYKGDEANNFFKLREDWWTENRETVWKAITCDAHGTYFR

Preich_024 HB3var169RTcupsB1

QQKYKDTGSSFTMCTMLARSFADIGDIIRGKDLFLGYNETDKEQKGKLQDNLKNIFAKIY

Q KYKD+GS+FTMCTMLARSFADIGDIIRGKDLF+GYN+ D+++K +LQ NLKNIFAKIY

QTKYKDSGSTFTMCTMLARSFADIGDIIRGKDLFIGYNQKDRKEKKQLQQNLKNIFAKIY

Preich_025 PFB1055c

DYTKKLYCSGNGYDCTKTIYKKGKLFIGEHCTKCSVWCRMYEKWIDNQKKEFLKQRKKY

DY + LYCSGNGYDCTKTIYKKGKL IGEHCT CSVWCR+YE WIDNQK EFLKQ++KY

DYKQNLYCSGNGYDCTKTIYKKGKLVIGEHCTNCSVWCRLYESWIDNQKLEFLKQKQKY

Preich_026 HB3var36

NYYKLREDWWTANRDQVWKAITCSDKLKGASYFRATCSDGNSKSQAR

NYY+LREDWW ANR+ VWKA+TCSD+L GASYF ATC DGNSKSQA+

NYYQLREDWWYANRETVWKAMTCSDELTGASYFHATCIDGNSKSQAK

Preich_032 PFC0005

ANRKQVWKAIRCGAPDDADYFIKNTCSEGRSSAEKKCRCIVGDVPTYFDYVPQYLRWFEEWAEDFCRLRKHKL

ANR+ VWKAIRC AP DADYFIK CS G++ + KCRCI VPTYFDYVPQYLRWFEEWAEDFCRLRKHKL

ANRETVWKAIRCSAPRDADYFIKTVCSGGKTPTQGKCRCIDFSVPTYFDYVPQYLRWFEEWAEDFCRLRKHKL

Preich_040 HB3var24

PNTDPPTYFDYVPQFLRWFEEWAEDFCRKKRKKLENVKKKCRGKYQGK

PNTDPPTYFDYVPQFLRWFEEW EDFCRKK+KKLENV+K+CRG+YQG+

PNTDPPTYFDYVPQFLRWFEEWGEDFCRKKKKKLENVQKQCRGEYQGE

Preich_040 PFL1955w

PNTDPPTYFDYVPQFLRWFEEWAEDFCRKKRKKLENVKKKCRG

PNTDPPTYFDYVPQ+LRWFEEWAEDFCRKK KKL +VKK+CRG

PNTDPPTYFDYVPQYLRWFEEWAEDFCRKKNKKLNDVKKQCRG

Preich_046 HB3var8

VPTYFDYVPQFLRWFEEWAEDFCTKRKHKLQNAINICRGMGDDGNKLYCDLNGFDCKRTAKGEERFVEGGGCKKCSVPCDHFVHWIDNQKEEFDKQKNKY

VPTYFDYVPQ+LRWFEEWAEDFCTKRKHKLQNAI ICRG DG KLYCDLNG+DC RTAKGE + C KCS+PCDHFVHWIDNQK+EF KQKN+Y

VPTYFDYVPQYLRWFEEWAEDFCTKRKHKLQNAIKICRGKDKDGKKLYCDLNGYDCTRTAKGENKRFSDSNCNKCSLPCDHFVHWIDNQKKEFLKQKNRY

Preich_046 PFC0005w

SVPTYFDYVPQFLRWFEEWAEDFCTKRKHKLQNAINICRGMGDDGNKLYCDLNGFDCKRTAKGEERFVEGGGCKKCSVPCDHFVHWIDNQKEEFDKQKNKY

SVPTYFDYVPQ+LRWFEEWAEDFC RKHKLQNA N CR DG KLYCDLNGFDC +TA+G+ ++ C +C CDHFVHWIDNQK+EF+KQKNKY

SVPTYFDYVPQYLRWFEEWAEDFCRLRKHKLQNAKNKCREKHKDGKKLYCDLNGFDCTQTARGKNKYKYEHDCIECYSSCDHFVHWIDNQKKEFEKQKNKY

Preich_046 HB3var8

CKKCSVPCDHFVHWIDNQKEEFDKQKNKY

C KCS+PCDHFVHWIDNQK+EF KQKN+Y

CNKCSLPCDHFVHWIDNQKKEFLKQKNRY

Preich_061 PF100406

QNAIKNCRGEKNDKYCDLNGYDCTKTIRGENKLV

QNAIKNCRGE N+KYCDLNGYDC KTIRG+ KL

QNAIKNCRGENNEKYCDLNGYDCEKTIRGKKKLF

Preich_061 PF140773_truncated

YRRLHLCDKNLEQIKPYQINNTHNLLADVCLAAKYEGESLVDKHKAYKETHNDFNTNICTVLARSFA

YRRLH+CD+NLE IKP QI +THNLL DV LAAK+EG+SLVDKHK YKETH D TNICTVLARSFA

YRRLHICDRNLELIKPDQITSTHNLLVDVLLAAKHEGKSLVDKHKKYKETHKD--TNICTVLARSFA

Preich_061 HB3var27

DVTIVPTYFDYVPQYLRWFEEWAEDFCRKKKKKL

DVTIVPTYFDYVPQYLRWFEEWAEDFCRKKKKKL

DVTIVPTYFDYVPQYLRWFEEWAEDFCRKKKKKL

Preich_078 PFL1970w

PCGNGSANGETVRFSKERDAKYDEKKIGCSNSEGACAPLRRLSLCNKNLENINNIHSDKARHNLLAEVCMAAKYEGESLKTYHEQYASLYPS

PCGNGSA+ RFSKER +YDEKKIGCSNSEGACAP RRL LCNKN N+N+ S KA+H+LL +VCMAAKYEGESLK YHEQY YPS

PCGNGSASASDKRFSKERVDEYDEKKIGCSNSEGACAPYRRLHLCNKNFPNMNSKDSSKAKHDLLVDVCMAAKYEGESLKVYHEQYEVQYPS

Preich_084 HB3var22

HNLENISDYEHINNHTLLADVCLAANFEGDSLRKHHGQHQLLNTDFHTNICTELARSFADIGDIVRGRDLYRGDNGKDKLEKNLKTIFGKIHENL

HNLENISD++HINN TLLADVCLAA FEG++L HGQHQL D H ICTELARSFADIGDI+RG+DLYR DN KDKLE NLKTIF +IHE L

HNLENISDFDHINNDTLLADVCLAAKFEGETLTTQHGQHQLTYPDSHYEICTELARSFADIGDIIRGKDLYRRDNKKDKLENNLKTIFKQIHEKL

Preich_084 PFC0005w

LARSFADIGDIVRGRDLYRGDNGKDKLEKNLKTIFGKIHE

LARSFADIGDI+RG+DLYRG+NGKDKLE+NLKTIFGKIHE

LARSFADIGDIIRGKDLYRGNNGKDKLEENLKTIFGKIHE

Preich_084 PFC1120c

KTCSNNKTDTDNKCHCVNGDPPTYFDYVPQYLRWFEEWAEDFCRKKDKKLKDLKKNCR

KTCSN+ +DT+ KC CV+ DPPTYFDYVPQYLRWFEEW E+FCRKK KKL+D+ K CR

KTCSNDTSDTNEKCRCVSTDPPTYFDYVPQYLRWFEEWTEEFCRKKKKKLEDVIKKCR

Preich_084 PFB1055c

PTYFDYVPQYLRWFEEWAEDFCRKKDKKLKDLKKNCR

PTYFDYVPQYLRWFEEWAEDFCRKK KKL+ L++ CR

PTYFDYVPQYLRWFEEWAEDFCRKKKKKLEKLEQQCR

Preich_102 HB3var16

RKKKLENAIKNCRQKEDKNKYCDLNGYDCEKTARGQNKFFPDSDCNKCSVACKPFVKWIDDKKQEFLKQKVKY

RK KL+NAIKNCR K+KYCDLNGY+CE+TA+ +NK FPDS+C+KCSVAC PFV WID++K+EF KQK KY

RKHKLQNAIKNCRTPNGKDKYCDLNGYNCEETAKKENKLFPDSECHKCSVACNPFVPWIDNQKKEFEKQKGKY

Preich_126 HB3var16

PCRNGTEKRFSDTEGAQCHSRKIRDSKGESEGACAPFRRLHLCDKNLQNISDFNKINNKHNLLLEVLLAAKYEGESITLDHPRY

PC NGTE+RFSDT+G++C RKI +KG++ GACAPFRRLHLCDKNLQNI++++KINN HNLLLEV LAAKYEG+SI+L +PRY

PCGNGTEERFSDTKGSECDDRKIEGNKGKTGGACAPFRRLHLCDKNLQNINNYDKINNTHNLLLEVCLAAKYEGQSISLYYPRY

Preich_129 PFL2665c

CGACAPYRRLHLCNHNLESIETNNYDSSNAKHKLLAEVCYAAKYEGDSLRKHHGKHKENNEGSRLCTELARSFADIGDIIRGKDLFRGNDEEKKKRKQLDEKLKDIFKKIYEELSKRNGAKQHYQDDNGGNFFKLREDW

CGACAPYRRLHLC+HNLESI+T NY+SSNAKH LLAEVC AAKYEG++L HGKH++ N S++CT LARSFADIGDI+RG+DLF GN +E +RK LDEKLK+IFK+I+ L+K++ Q Y D+NGGNFFKLREDW

CGACAPYRRLHLCHHNLESIQTKNYNSSNAKHDLLAEVCMAAKYEGETLTTEHGKHQQTNNDSQICTVLARSFADIGDIVRGRDLFHGNPQESAQRKVLDEKLKEIFKEIHSGLTKKDA--QTYYDENGGNFFKLREDW

Preich_133 PF080103

PCKDRPDVRFSDEYGGQCTDRKIKGNNEGTGGACAPLRRLFLCDHHLSHMKAEKIDNTHNLLAEVCLAAKHEGQSIKGYHDKYRSKYIDTNSQLCTVLARSFADIGDIIRGKDLFIGYDENDRKEKKKLEKSLKNIFAKIYNNLI

PCKDRP+VRFSDEYGGQCTD KIKGN + GGACAP RRLFLCD HLSHMKAEKI+N HNLL EVCLAAK+EG+S+KGYHDKY + Y D+ SQLCTVLARSFADIGDIIRGKDLFIGYD+ DR +KKKL+ SLKNIF IYN L

PCKDRPEVRFSDEYGGQCTDSKIKGNEDNKGGACAPFRRLFLCDQHLSHMKAEKINNKHNLLLEVCLAAKYEGESLKGYHDKYNATYSDSRSQLCTVLARSFADIGDIIRGKDLFIGYDKKDRVQKKKLQDSLKNIFGNIYNELT
